# Ancient genomes reveal basal Asian ancestries and dynamic population interactions over time on the southern Tibetan Plateau

**DOI:** 10.1016/j.isci.2025.113676

**Published:** 2025-10-03

**Authors:** Jingkun Ran, Yichen Liu, Shargan Wangdue, Xiaoyan Yang, Tianyi Wang, Yu Gao, Peng Cao, Yan Tong, Qingyan Dai, Songtao Chen, Han Shi, Feng Liu, Xiaotian Feng, Yazhong Li, Fahu Chen, Qiaomei Fu

**Affiliations:** 1 Group of Alpine Paleoecology and Human Adaptation (ALPHA), State Key Laboratory of Tibetan Plateau Earth System, Resources and Environment (TPESRE), Institute of Tibetan Plateau Research, Chinese Academy of Sciences, Beijing, China; 2 Key Laboratory of Vertebrate Evolution and Human Origins, Institute of Vertebrate Paleontology and Paleoanthropology, Chinese Academy of Sciences, Beijing, China; 3 University of Chinese Academy of Sciences, Beijing, China; 4 Tibetan Institute of the Preservation of Cultural Relics, Lhasa, China; 5 Key Scientific Research Base of Bioarchaeology in Cold and Arid Regions (National Cultural Heritage Administration), College of Earth and Environmental Sciences, Lanzhou University, Lanzhou, China; 6 National Centre for Archaeology, Beijing, China

**Keywords:** Genomics, Human geography, Paleogenetics, Population

## Abstract

The southern Tibetan Plateau is a key region for the settlement and flourishing of Tibetan populations, but the long-term population dynamics in this area remain poorly understood. By analyzing 16 newly sequenced genomes from the Mabu Co site (4400 masl), we reconstructed the population history on the southern Tibetan Plateau spanning 4,400–3,500 BP. While the local southern plateau ancestry was maintained over a millennium, genetic diversity within these populations was observed over time. We revealed multiple admixtures, including Basal Asian Xingyi-related ancestry linked to hunter-gatherer populations from southwestern China, and provided the first evidence of this ancestry influencing the ancient southern Tibetan Plateau populations again after 4,000 BP. Moreover, mtDNA and Y haplogroups reveal that individuals exhibit greater maternal genetic diversity alongside restricted paternal lineage conservation. This study provides a higher-resolution population history in the ultra-highlands, contributing to understanding human adaptation to extreme environments.

## Highlights

• 16 new ancient genomes from the southern Tibetan Plateau (4400–3500 BP)
• Continuity and divergence of local plateau ancestry over a millennium
• Detected Basal Asian ancestry signals from southwestern China
• Revealed sex-specific patterns and EPAS1 shifts in adaptation and demography

## Graphical abstract

**Figure:**
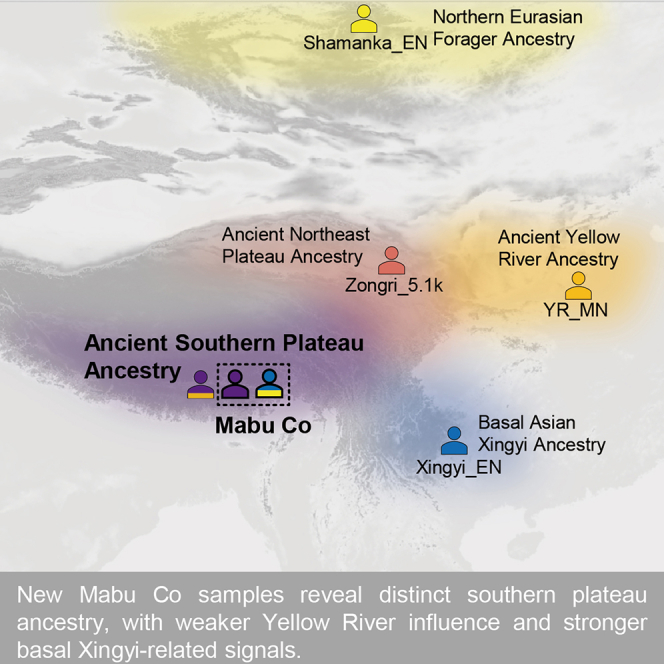

## Introduction

The prehistory of ancient humans on the Tibetan Plateau contributes to the understanding of human evolution in extreme environments. Denisovans were present on the plateau as early as 160,000 years ago and possibly persisted until around 40,000 years ago,^1^^,^^2^ potentially overlapping with early modern human ancestors who may have arrived at the plateau prior to the Last Glacial Maximum (LGM).^3^ Subsequently, archaeological data suggest that hunter-gatherer groups with distinct microblade technology arrived on the northeastern plateau margins ∼14,000–10,000 BP.^4^^,^^5^ Similarly, Tibetan populations were estimated to be divergent from the lowland East Asians ∼15,000–9,000 BP using present-day genetic data.^6^ Agriculture techniques and related Sino-Tibetan languages were later introduced to the Tibetan Plateau, likely facilitated by the expansion of millet farmers associated with the Yangshao Culture from the Upper and Middle Yellow River regions around 7,000–5,000 BP, while the process of multiple migrations is controversial.^7^^,^^8^^,^^9^ This led to the establishment of an extensive millet-based agricultural system across the plateau.^10^ However, hunting-gathering activities persisted among Tibetan populations until the mid-Holocene (∼4,000 BP).^11^^,^^12^ After 4,000 BP, a significant agricultural transition occurred with the introduction of barley- and wheat-based farming systems alongside domesticated sheep and goats.^13^^,^^14^ Concurrently, bronze technology arrived alongside these developments, though its stratigraphic association with iron artifacts has led scholars to term this phase the “Early Metal Age” that persisted until the 7th century CE.^15^^,^^16^

Ancient genomes can provide high-resolution evidence on the plateau population history during this period.^17^^,^^18^^,^^19^^,^^20^^,^^21^^,^^22^ For early ancient Tibetans (5100–2500 BP),^20^ genomic analyses suggest that their ancestry can be modeled as approximately 80% northern East Asian ancestry and 20% derived from recently reported Xingyi_EN related ancestry.^23^ This Xingyi_EN individual, a 7,100-year-old from central Yunnan, was denoted as Basal Asian Xingyi-related ancestry within the Asian branch, likely separating from other known Asian diverged lineages (e.g., Tianyuan, Onge) at least 40,000 years ago.^23^ In addition to this complex ancestral makeup, previous studies have revealed three major regional genetic lineages within the plateau.^20^^,^^22^ One of the earliest is represented by individuals from the northeastern Zongri site (∼5,100 BP), which currently provides the oldest available genetic evidence from the Tibetan Plateau.^20^ However, considering most present-day Tibetans living above 3,000 m above sea level (masl), the 2,820 masl Zongri is unlikely to represent the entire plateau, and our understanding of ancient Tibetans living on the hinterland remains limited.

The southern Tibetan Plateau, with an average altitude higher than 4,000 masl, has been a core living region for past plateau dwellers. The midstream valleys of the Yarlung Tsangpo River are characterized by relatively warm climates since the Holocene compared to the northern and western Plateau.^24^ Genetic evidence from present-day Tibetans suggests the southern Tibetan Plateau is likely a refuge for early Paleolithic settlers.^25^^,^^26^ Ancient DNA studies reveal that individuals dating back to around 3,000 years ago from Shannan, Ngari, and Nepal (represented by the Shanan3k, GBSL, and Lubrak populations) exhibited a consistent genetic profile, referred to as the southern Plateau ancestry.^19^^,^^20^^,^^22^ Indeed, the southern plateau ancestry has played an important role in shaping the genetics of the population from surrounding regions and present-day Tibetans in Lhasa, Shannan and Shigatze.^20^ More importantly, the Neolithic covers a crucial period when indigenous plateau hunter-gatherers dynamically interacted with the lowland East Asia farmers, such as the millet agriculture related Proto-Sino-Tibetan speakers.^27^ Additionally, cultural connections between Tibetans and Central Asia/Egyptian might appear before 3,500 BP.^28^^,^^29^ Furthermore, the introduction of cattle and sheep^12^ as well as changes in the subsistence economy^30^^,^^31^ suggest extensive communications in culture and technology during this period.^32^^,^^33^ However, due to the limited geographic and temporal coverage of previous genetic data, current understandings of the southern Tibetan Plateau populations remain fragmented, especially in the late Neolithic period, rendering the related genetic and cultural exchanges unclear.

**Figure 1:**
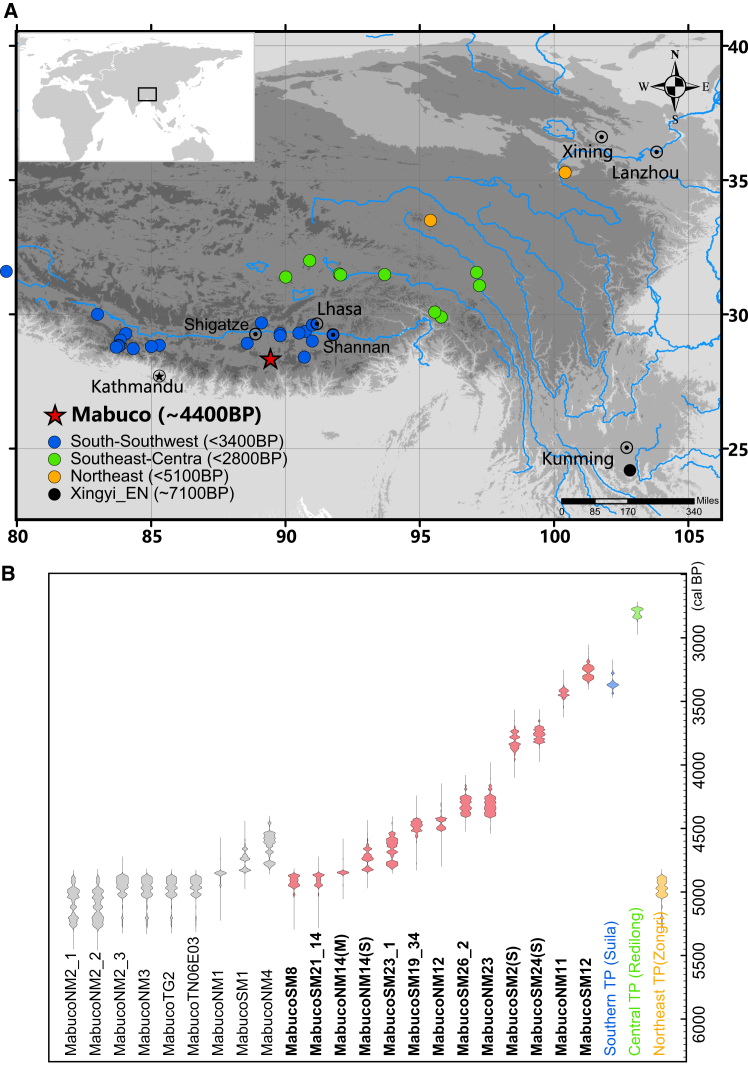
Location of the Mabu Co site and radiocarbon dating results of Mabu Co human bones (A) Mabu Co site highlight as the red stars, and the previously published sites with paleogenomics on the three different plateau regions (southern, central, and northeast plateau) were shown as blue, green, and yellow circles. Details of previously published sites are provided in Table S14. (B) The probability density of calibrated dating result, details in Table S1. The red means new Mabu Co samples, the gray means previously published Mabu Co data, and the blue, green, and yellow refer to the earliest dating result of archaeological sites with paleogenomics (named in parentheses) in the three plateau regions.

To gain a clearer understanding of the early genetic history of the southern Tibetan Plateau populations and their connections with surrounding populations, we obtained 16 new ancient genomes from the Mabu Co site on the southern Tibetan Plateau (Figure 1A), spanning from 4,400 to 3,500 BP. This site is situated near the Mabu Co Lake, which is connected to the Nianchu River, a tributary of the middle reaches of the Yalung Tsangpo River. This is the oldest Neolithic site in the hinterland of the plateau from which ancient human genomes have been recovered. It is located at an altitude of 4,446 masl and represents a lake-centered lifestyle of indigenous fishing-hunting populations before 4,000 BP.^11^ Through analyzing the data from Mabu Co, we aim to investigate long-term genetic dynamics over a thousand years, thereby exploring the genetic makeup of ancient southern Tibetan Plateau populations as well as the interactions with lowland populations.

## Results

We generated genome-wide data at ∼1.2 million single-nucleotide polymorphisms (SNPs) for 16 new sampling human specimens from the Mabu Co site, as the earliest Neolithic site on the southern Tibetan Plateau, where human bones have been excavated. The direct radiocarbon dating for human bones ranges from 5,023 to 3,347 BP (Figure 1B). As the site is close to a lake (Mabu Co) and fish is part of the diet of these ancient individuals,^11^ direct carbon dating is likely to be affected by the carbon reservoir effect (i.e., intake of carbon from the water system can bias carbon date estimation that mostly based on atmospheric data^34^). Therefore, we employed a more conservative estimation and grouped the samples into Early (∼4,400 BP), Middle (∼4,000 BP) and Late (∼3,500 BP) periods based on the chronology of terrestrial animals and plants from the same stratigraphic level (Figure S1; Table S1).

Across the 16 individuals, the sequencing depth for “1240k” SNPs ranged from 0.22 to 3.35×, and the number of SNPs ranged from 231,354 to 848,263. We combined the previously published 9 Mabu Co individuals^11^ for kinship analysis to determine whether any individuals were related to each other. Of the 25 individuals, 3 paired kinships (first- and second-degree) were found, and for each kinship group, the individuals with AMS ^14^C dating age and high SNPs count were retained (Table S1) to reduce potential biases in population genetic analyses introduced by closely related individuals. The total 22 Mabu Co individuals were combined with published present-day and ancient populations from the whole Tibetan Plateau and surrounding regions, especially those from East Asia, for population genomics analysis (STAR Methods).

### The genetic origin of the earliest ancient southern Tibetan plateau populations at 4,400 BP

**Figure 2:**
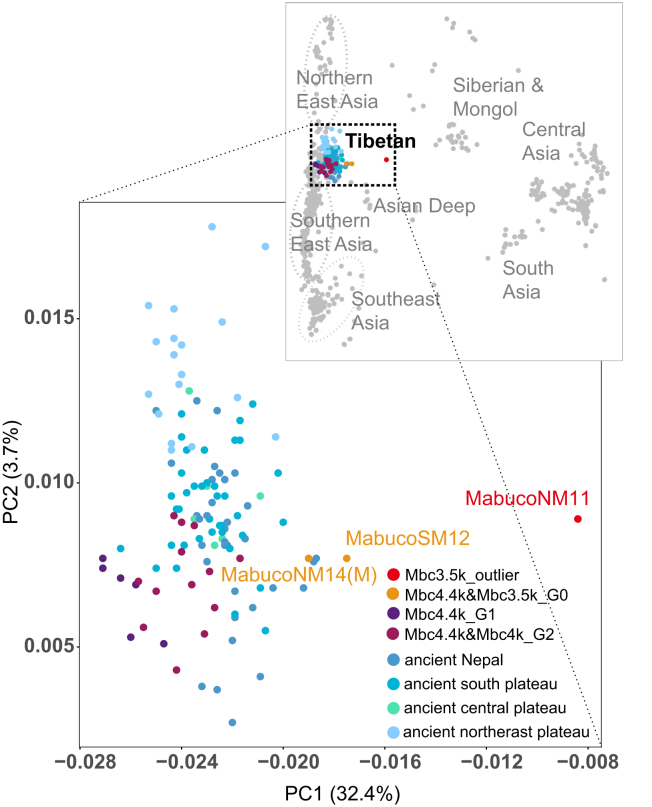
Genetic affiliation and relationships among individuals revealed by PCA Ancient Tibetan Plateau individuals are projected onto the principal components calculated by present-day Central and East Asian populations, with a zoomed-in PCA view focusing on ancient Tibetan populations and the Mabu Co individuals labeled with sample IDs. Points of different shades of blue represent published ancient populations from Nepal and three regions of the Tibetan Plateau (northeast, central, and southern regions).

To explore the genetic characteristics of the Mabu Co individuals, we conducted principal component analysis (PCA), projecting the ancient samples onto two sets of present-day Eurasian and East Asian populations (Figure 2; Figure S2). The majority of Mabu Co individuals clustered with both present-day and ancient Tibetan Plateau populations, except for one outlier Mbc3.5k_outlier (MabucoNM11, Male). Most newly sampled individuals align closely with previously published Mabu Co populations from the early periods, clustering near the southern plateau population groups.

**Figure 3:**
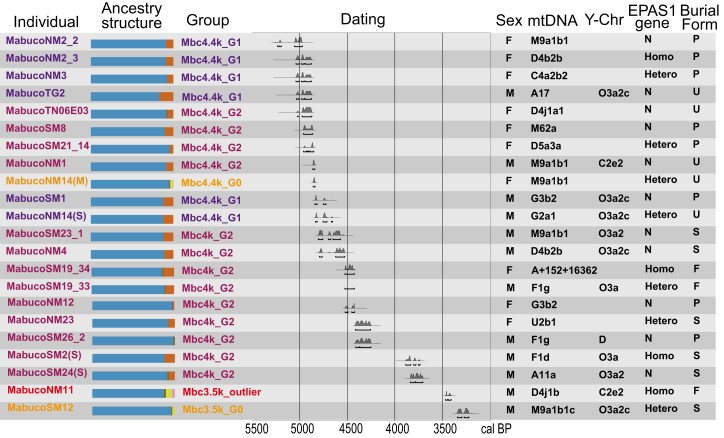
Summary of genetic characteristics, groupings, dating, parental haplogroups, adaptive genes, and burial forms for each individual Ancestry structure column shows the results of ADMIXTURE analysis (K = 5), where blue and brown represent the general high-altitude and low-altitude East Asian components, besides, the bright yellow color represents the Anatolia_N agricultural population from the Fertile Crescent, and the pink color denotes the ancient Papuan-related ancestry from Island Southeast Asia, respectively, and the complete and detailed ADMIXTURE results can be seen in Figure S3. Burial Form column categorizes burial types: P represents prone burials, S represents secondary burials, F represents fetal position burials, and U indicates unknown burial forms.

Using PCA, f3, and f4 statistics based on genetic differences between individuals, we genetically grouped the 22 individuals from the Mabu Co site (Mbc). In the early period (∼4400 BP, *n* = 13), individuals can be grouped into 3 genetically distinct groups (G0, G1, and G2), mostly based on their affinity to East Asians. The previously published Mbc4.4k_G1 and Mbc4.4k_G2 possess the local southern plateau ancestry (represented by Shannan3k population^11^) admixed with varied proportions of East Asian ancestry (G1: 66%–71% local southern plateau ancestry +29%–34% East Asian; G2: 100% local plateau ancestry).^26^ In contrast, the newly sampled contemporary individuals (Mbc4.4k_G0 and Mbc3.5k_G0) clustered separately from G1 and G2 along the PC1, and distant from southern and northern East Asians (Figure S2). Based on this pattern, supported by both f3 and f4 statistics among Mabu Co individuals (Figure S3; Figure S4), they were designated as the G0 group. Specifically, consistent with the PCA results, f3 statistics among individuals further confirmed the existence of genetic differences between both the Mbc4.4k_G0 group and the outlier individuals (Figure S3). The magnitude of genetic differences between individuals was further revealed in pairwise f4 analyses. While most pairs of individuals within the G1 and G2 group lacked significant genetic differences, both the G0 group and the outlier individuals exhibited generally large genetic difference values relative to other individuals (Figure S4; Table S2). Similar results are observed in ADMIXTURE-based ancestral modeling (Figure 3; Figure S5), where clusters G1 and G2 were primarily characterized by the indigenous Tibetan Plateau ancestry (blue component) with minor East Asian lowland-associated ancestral components (brown components), while G0 and the outlier individuals exhibited increased ancestral complexity (bright yellow and pink components).

**Figure 4:**
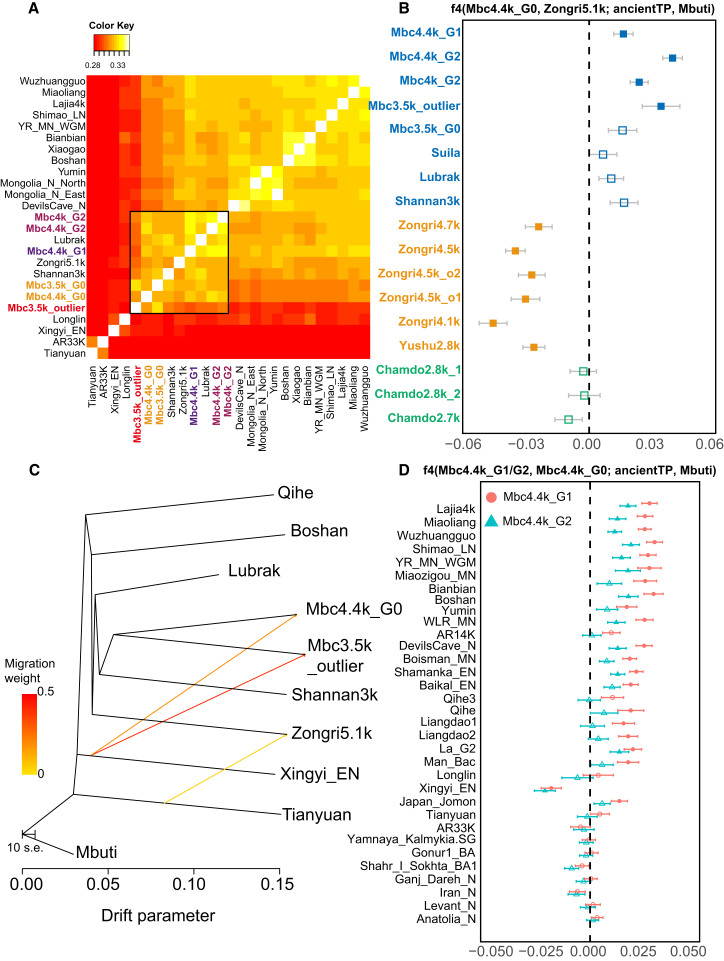
Population structure and genetic profiles of Mabu Co populations (A) Heatmap of outgroup-f3 statistics for different groups, including ancient Tibetan Plateau populations, Northern East Asian populations, and Deep East Asian populations. The ancient Mabu Co populations cluster closely with other ancient Tibetan Plateau populations in the outgroup-f3 analysis. (B) Results of f4(Mbc4.4k_G0, Zongri5.1k; ancient TP, Mbuti), where ancient TP includes ancient populations from the southern (blue), northeastern (yellow), and central (green) regions of the Tibetan Plateau. Positive f4 values indicate a closer genetic relationship with the ancient southern Tibetan Plateau populations (Mbc4.4k_G0), while negative f4 values suggest a closer affinity to northeastern ancient populations (Zongri5.1k). Filled squares represent significant results, whereas open squares represent non-significant results (|Z| < 3). Error bars indicate ±2 SE. (C) TreeMix analysis of ancient Tibetan Plateau populations within the Asian context. The maximum likelihood tree reveals a genetic bifurcation between southern (Mbc, Lubrak, Shannan3k) and northeastern (Zongri5.1k) Tibetan lineages, consistent with regional genetic differentiation, and inferred migration edges indicate a gene flow event from a Basal Asian Xingyi-related source into the Mbc4.4k_G0 group, as well as a stronger gene flow into the Mbc3.5k_outlier group. (D) Results of f4(Mbc4.4k_G1/G2, Mbc4.4k_G0; Y, Mbuti), where Y includes non-plateau populations from East Eurasia and Central Asia. Circles represent Mbc4.4k_G1, and triangles represent Mbc4.4k_G2. Filled shapes indicate significant results (|Z| > 3), and open shapes represent non-significant results (|Z| < 3). Error bars indicate ±2 SE. The analysis indicates that both G1 and G2 have more genetic connections to low-altitude East Asian populations than G0, with G1 exhibiting stronger associations.

As the newly identified Mbc4.4k_G0 group showed slightly different genetic affinities to Tibetan Plateau genetic components, we conducted comprehensive investigations into its genetic profile. This analysis builds upon previously established genetic substructures of the Tibetan Plateau, which identified distinct southern and northeastern ancestries. By statistically summarizing allele sharing using f4-statistics of the form f4(Mbc4.4k_G0, Zongri5.1k; ancientTP, Outgroup), we identified distinct ancestry profiles for these two groups within the plateau populations (Figure 4B), and this pattern is consistent with results from f4-ratio statistics, revealing that southern individuals (e.g., Shannan3k, Lubrak) are more closely related to Mbc4.4k_G0 (Figure S6). These findings are further supported by TreeMix analysis, which reveals a clear genetic bifurcation between the southern and northeastern lineages on the Plateau (Figure 4C), confirming that Mbc4.4k_G0 and Zongri5.1k serve as the early ancient southern and northeastern population ancestry on the Tibetan Plateau, respectively. In the context of the East Asian populations, the genetic similarity between Mbc4.4k_G0 and Zongri5.1k is verified by additional f4 (Mbc4.4k_G0, SEA/DEEP; Zongri5.1k, Mbuti) > 0, the majority Z obtained from which are greater than 3.0 (3.0 < Z < 13.1), confirming Mbc4.4k_G0 and Zongri5.1k shares more genetic similarities compared to the tested SEA/DEEP populations. Consistently, Mbc4.4k_G0 and Zongri5.1k can be modeled as primary sources for each other in qpAdm analysis (Table S3), confirming the high affinity between the two. Nevertheless, a subtle genetic difference between Mbc4.4k_G0 and Zongri5.1k is still observed in f4(Mbc4.4k_G0, Zongri5.1k; Lajia4k/Shamanka_EN/Baikal_EN/, Mbuti) < 0 (−4.8 < Z < −3.1) (Figure S7), as also supported by non-significant results with North East Asian as f4 (Zongri5.1k/Mbc4.4k_G0, NEA; Mbc4.4k_G0/Zongri5.1k, Mbuti) ∼0 (−0.9 < Z < 2.3) (Table S3), suggesting that the Mbc4.4k_G0 and Zongri5.1k are not direct sister groups. TreeMix analysis suggests a possible signal of gene flow from Basal Asian Xingyi-related ancestry into Mbc4.4k_G0 (Figure 4C). In line with this, 2-way qpAdm modeling suggests that the Zongri5.1k could be modeled predominantly as Mbc4.4k_G0 ancestry, with a small amount of NEA component (15.2%–20.1%), while Mbc4.4k_G0 could be modeled as mostly Zongri5.1k ancestry, admixed with a small proportion of Xingyi_EN (∼6.2%, *p*-value:0.1105) (Table S4). Consistent with this, qpGraph modeling yields one plausible reconstruction, in which approximately 36% of the ancestry in Mbc4.4k_G0 is modeled as deriving from Basal Asian Xingyi-related ancestry, while the remaining ∼64% comes from northern lowland East Asians, consistent with other early Plateau populations such as Zongri5.1k (Figure S8). Using a genealogy-based chromosome painting approach on imputed genetic data, we consistently detected a high proportion of ancestry related to Xingyi_EN in the G0 group (Figure S9). In summary, while the Mabu Co individuals share a local ancestry with other early Tibetan populations, genetic differentiation introduced by Basal Asian Xingyi-related ancestry is observed in a proportion of Mabu Co populations compared with Zongri5.1k.

**Figure 5:**
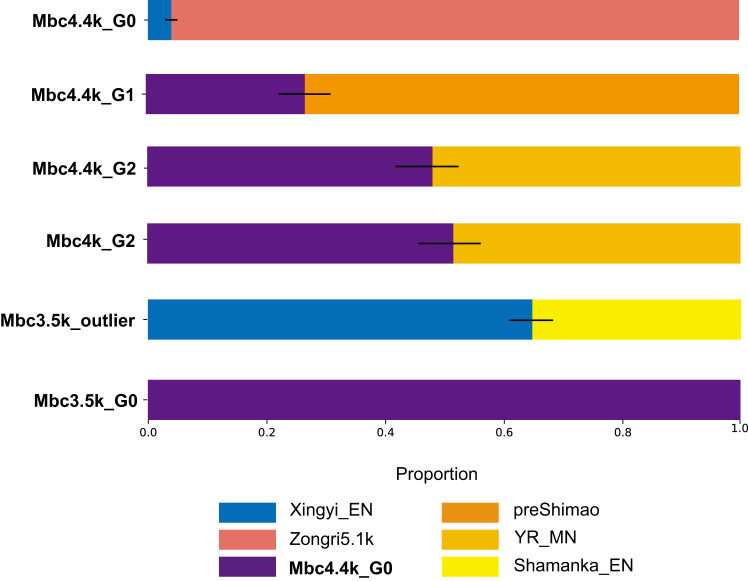
Genetic ancestry continuity and interactions in Mabu Co populations Details regarding the left and right populations used in the modeling, as well as the rotation strategy for qpAdm, are described in the STAR Methods. Admixture proportions for the early periods are detailed in Table S4, while those for the middle and late periods are provided in Table S8. Error bars indicate ±2 SE. For Mbc4.4k_G0, although it can be one-way modeled using Zongri5.1k, indicating a sister-group relationship under known ancient East Asian populations, genetic differences revealed by f4 suggest that Mbc4.4k_G0 and Zongri5.1k have subtle differences and represent distinct ancestral components of the northeastern and southern Tibetan Plateau populations. Therefore, a two-way admixture model is used in the figure to better represent both their relationship and differences. For the genetically consistent groups Mbc4.4k_G2 and Mbc4k_G2, while Mbc4.4k_G2 can one-way model Mbc4k_G2, we used the same two-way model for both groups in the figure to show their genetic similarity due to complex color schemes.

Leveraging the genetic profile of Mbc4.4k_G0 from the southern plateau, we can shed new light on the genetic landscape of early plateau populations. The relative affinity of Mbc4.4k_G0 to ancient southern Tibetan Plateau populations provides a baseline for comparison. Compared with Mbc4.4k_G0, Mbc4.4k_G1 and Mbc4.4k_G2 groups exhibit substantial gene flow from lowland East Asian populations, as evidenced by significantly positive f4 (Mbc4.4k_G1/Mbc4.4k_G2, Mbc4.4k_G0; NEA/SEA, Mbuti) > 0 (most Z > 3) (Figure 3D; Table S5). This indicates the possibility of partial external gene flow primarily affecting the Mbc4.4k_G1 and Mbc4.4k_G2 groups. Consistently, different admixture levels of Yellow River-related components are obtained through qpAdm models, in which both G1 and G2 can be two-way modeled by G0 and populations related to pre-Shimao or Middle Neolithic Yellow River (YR_MN) farmers populations, while the proportion of latter in G1 and G2 are 73.2% and 48.1%, respectively (Figure 5; Table S4). The admixture can be dated to 19 ± 6 (Z: 2.88, normalized root-mean-square deviation [NRMSD]: 0.167) generations (assuming 29 years per generation, 364–700 years) for G1 and 11 ± 2 (Z: 5.09, NRMSD: 0.155) generations (252–364 years) for G2 (Figure S10; Table S6). Therefore, the indigenous Mabu Co population (whose genetics are represented by G0) was introgressed by lowland East Asians to Mabu Co populations 252 to 700 years earlier than the site, as 4,652-5,100 BP, and the introgression impacted the G1 and G2 differently, which introduced the fine genetic divergence to Mabu Co populations.

### Population dynamics and genetic continuity on the southern Tibetan plateau from 4,400 BP to 3,500 BP

Previous studies are mostly concentrated on a large number of archaeological sites scattered across the Tibetan plateau, providing a broad genetic overview of ancient southern Tibetans, while the temporal genetic dynamics within a single continuous settlement remain unclear. To this end, we examined the genetics of Mabu Co populations from 4,400 to 3,500 BP and tracked their genetic changes over time. Specifically, these individuals are categorized into three time periods: early period (∼4,400 BP, 11 individuals), middle period (∼4,000 BP, 9 individuals), and late period (∼3,500 BP, 2 individuals).

We next sought to determine whether genetic continuity characterized the primary population lineage at Mabu Co between the early and middle periods. Focusing on the population genetic profile in middle period (∼4,000 BP), we observed that the major genetic component identified as Mbc4k_G2 exhibited strong affinity to the early period Mbc4.4k_G2 group, as supported by the close clustering in the individual pair f3 analysis (Figure S3) and no significantly different level of shared alleles detected by f4(ind., ind.; world, Mbuti) with mostly no significant genetic differences observed (Figure S4). Furthermore, population-level f4 tests confirmed the genetic similarity between the early Mbc4.4k_G2 and middle Mbc4k_G2 populations, evidenced by f4(Mbc4k_G2, Mbc4.4k_G2; other, Mbuti) ∼0 (−0.6 < Z < 2.5) (Figure S11; Table S7). This continuity was further supported by qpAdm models, which successfully modeled Mbc4.4k_G2 as a single source for Mbc4k_G2 (*p* value = 0.4143; Table S8). As a result, Mbc4.4k_G2 and Mbc4k_G2 represent a genetically continuous southern plateau lineage at Mabu Co spanning from 4400 to 4000 BP.

Given the observed continuity within Mabu Co, we then explored the broader regional presence of this dominant G2 lineage after ∼3,500 BP. The G2 component was not the major contributor in the two sampled late-period Mabu Co individuals. However, this ancestry persisted prominently in contemporaneous populations across the southern Tibetan Plateau, notably at Shannan3k and Lubrak. Consistent with the no significant differences reflected in f4 (Mbc4.4k_G2/Mbc4k_G2, Shannan3k/Lubrak; other, Mbuti) ∼0 (−2.8 < Z < 2.5) (Table S7), the G2 lineage also likely served as a single source for Shannan3k and Lubrak by qpAdm (Table S8).

The late period at Mabu Co (∼3,500 BP) revealed the presence of individuals with distinct genetic profiles. One individual, SM12(Mbc3.5k_G0), aligns closely with the earlier Mbc4.4k_G0 lineage (Figure 3; Figures S3 and S4). Strong genetic similarity between Mbc3.5k_G0 and Mbc4.4k_G0 is evidenced by non-divergent f4-statistic f4(Mbc3.5k_G0, Mbc4.4k_G0; other, Mbuti) ∼0 and ancestral modeling showing Mbc3.5k_G0 derives predominantly from Mbc4.4k_G0 (*p* *value* = 0.171; Table S8). Furthermore, to account for possible confounding factors arising from additional later Basal Asian Xingyi-related ancestry contributions to Mbc3.5k_G0, we conducted DATES analyses to determine whether a recent admixture has occurred. DATES modeling of Mbc3.5k_G0 tentatively estimated a proximal admixture time frame between Mbc4.4k_G2 and Xingyi_EN-related components, but validation metrics fell below reliability thresholds, failing to meet minimum standards for temporal resolution (Z > 2; nrmsd<0.6) (Table S6). These statistically underpowered results preclude a definitive interpretation of Mbc3.5k_G0 as a recently admixed lineage.

In summary, the genetic dynamics of the Mabu Co populations from 4,400 to 3,500 BP reveal a pattern of continuity in the middle period and genetic differentiation in the early and late periods. During the early period, this Mbc4.4k_G2 lineage became a defining feature of the southern plateau genetic landscape, compared with the more lowland East Asian-influenced Mbc4.4k_G1 and much Basal Asian Xingyi_EN-related Mbc4.4k_G0. The dominant lineage, which demonstrated genetic stability to Mbc4k_G2, also persisted into surrounding ancient Shannan3k and Lubrak populations following 3,500 BP. Further solidifying the dominance of ancient southern plateau ancestry in shaping regional genetic landscapes. However, by 3,500 BP, genetic diversity increased again, marked by the appearance of an outlier (NM11) and a resurgence of plateau-pure ancestry in SM12 (Mbc3.5k_G0). These changes suggest that the genetic landscape of the southern plateau evolved through both continuity and genetic variation over time.

### Interaction between hunter-gatherers from southwestern China and late-period Mabu Co populations

In addition to the main southern Tibetan Plateau population group from the late period, one individual, NM11, stands out as an outlier, genetically distinct from the main Tibetan group. This individual displayed a unique genetic ancestry tied deeply to the Tibetan Plateau (Figures 2 and 3), as well as evidence of interaction with hunter-gatherer groups from southwestern China. Compared to the main Mabu Co population, NM11 (Mbc3.5k_outlier) exhibited strong genetic affinities to the Xingyi_EN lineage. This connection is supported by f4(Mbc3.5k_outlier, other; Xingyi_EN, Mbuti) > 0 (mostly Z > 3.0, except Zongri5.1k, Mbc4.4k_G0, Mbc3.5k_G0 ∼0; Table S9).

To further investigate its genetic composition, qpAdm was employed to model Mbc3.5k_outlier using older Mabu Co populations as potential sources. However, none of the earlier Mabu Co groups or Zongri5.1k could serve as a single source for Mbc3.5k_outlier, nor could models combining earlier Mabu Co populations with other ancestry components pass, suggesting the introduction of an external population. The only successful qpAdm model proposed that Mbc3.5k_outlier was composed of 64.8% Xingyi_EN and 35.2% Shamanka_EN (*p*-value: 0.0505, std error: 0.037, Table S8), reflecting the ancestral connection to ancient hunter-gatherer populations from southwestern China, coupled with a Northern East Asian component.

To further understand the timing of the admixture events, DATES analysis was applied to estimate the time of Xingyi_EN related admixture in Mbc3.5k_outlier. The results indicate admixture between Northern East Asian and Xingyi_EN lineages occurred 7,347–8,847 BP (standard error: ±2592 years, Z > 2, nrmsd <0.7; Figure S10; Table S10). This date is closely aligned with the age of Xingyi_EN (∼7ka), suggesting that the admixture event occurred in a population closely related the Basal Asian Xingyi-related ancestry.

### Genetic and adaptive differences between paternal and maternal inheritance at the Mabu Co site

Considering the unique, diverse culture and descent practice in ancient Tibetan populations,^22^ we analyzed mitochondrial (mtDNA) and Y chromosome haplogroups to investigate potential sex-specific demographic processes reflected through differentiated maternal and paternal lineage histories. The ancient mitochondrial DNA (mtDNA) results from the Mabu Co site, dating to 4,400 BP-3500 BP, reveal a diverse range of haplogroups commonly associated with East Asian populations (Figure 3; Table S1; Table S11). The most dominant haplogroup, M9a1b1, found in six individuals, is a well-established lineage in both ancient and modern East Asian populations, particularly prevalent on the Tibetan Plateau and in southern China.^21^^,^^35^^,^^36^ Other haplogroups, such as D4b2b, D4j1a1, D5, and C4a2, are typically associated with northern and Central Asian populations,^21^^,^^37^ but are also present in high-altitude regions such as Tibet, indicating a genetic continuity from ancient to the present-day plateau inhabitants. The presence of haplogroups A11a and A17, primarily associated with present-day Northeast Asian populations, particularly Siberia and northern China,^26^^,^^38^ reflects the northern origin in shaping the plateau maternal inheritance during the Late Pleistocene or early Holocene, and haplogroup F1g, more common in present-day East Asians and Southeast Asians,^39^ likely reflects the interactions from southeastern regions. The identification of less frequent haplogroups such as G3b2 and U2b1^21^ further emphasizes the genetic complexity of the ancient populations at Mabu Co. Such diversity in mtDNA suggests extensive female-driven genetic interactions with surrounding regions in the Mabu Co populations, despite the constraints of the high-altitude environment.

Compared to the maternal, the paternally inherited Y chromosome in the Mabu Co site is dominated by two haplogroups (O3a and C2e2) (Figure 3; Figure S12; Table S1), which are significantly less diverse and show signs of influence from lowland populations. The dominant haplogroup, O3a2c (now reclassified under O2-M122), was found in nine individuals. This haplogroup is widely associated with early agricultural populations in East Asia, including Han Chinese, and originated approximately 5,000 to 7,000 years ago, and has been linked to the peopling of the Tibetan Plateau.^40^^,^^41^^,^^42^^,^^43^ Haplogroup C2e2, found in two individuals, is part of a broader C lineage common in present-day northern and central Asia and also among modern Tibetan populations, reflecting gene flow between the plateau and northern regions.^44^ The presence of haplogroup D in one individual is particularly significant, as it is closely associated with deeply diverged ancient high-altitude populations before LGM, including modern Tibetan and Andamanese groups, underscoring the long-term occupation of the local Tibetans in the plateau. Interestingly, while the YR-related mitochondrial haplotypes (M9a1b1 (3/12) and D4j1 (2/12)) only represent 5/12 of the males, the YR-related Y-chromosome haplotype O3a dominant the Mabu Co individuals (9/12), with only 3 exceptions out of 12 individuals. This observation may suggest the genetic influence observed in Mabu Co individuals from the lowland YR populations could have been predominantly introduced through male lineages during the sampled period.

*The EPAS1* gene is a hypoxia-related gene involved in oxygen-sensing pathways, with adaptive variants under positive selection in Tibetans due to the beneficial effects of its protein product on high-altitude adaptation.^45^^,^^46^^,^^47^ Here, we found that, among the 10 Mabu Co females, 6 of them carry the adaptive *EPAS1* gene. The frequencies of *EPAS1* carriers among females in the early period were 4/7, and 2/3 in the middle period. Conversely, among the 12 male individuals, 5 carried the *EPAS1* gene, and the frequencies of *EPAS1* carriers among males were 1/4 in the early period, 2/6 in the middle period, and 2/2 in the late period (Figure 3; Table S12).

## Discussion

Through the genomes of 16 ancient individuals on the southern Tibetan Plateau covering ∼1,000 years, we were able to reconstruct the population genomics of the Mabu Co people, trace the ancient lineage that contributed to the local Tibetans, track the fine population interactions over time, and explore the potential sex-specific demographic processes.

Firstly, we identified an individual (Mbc3.5k_outlier), ∼2/3 genetic components can be linked to a Basal Asian Xingyi-related ancestry from southwestern China. This hunter-gatherer ancestry existed in Yunnan at ∼7,000 BP, and here we observed it later reappeared in the Tibetan Plateau. This suggests population interactions between ancient Tibetans and groups carrying this hunter-gatherer ancestry, likely mediated through the Tibetan-Yi Corridor.^48^ Supporting this hypothesis, recent findings from the high-altitude Su-re site revealed stone tool technologies linked to Southeast Asian traditions,^49^ further substantiating connections between Tibet and Southwest East Asia since the Paleolithic period. These findings suggest that early population interactions and cultural exchanges on the Tibetan Plateau were more active and complex than previously assumed, which has previously emphasized Late Neolithic north-south exchanges along the plateau’s eastern edge,^50^ and sampling of ancient populations that are representative of the ancient Tibetan-Yi Corridor ancestry would be key to explaining such population history. Additionally, this study provides the first ancient genetic evidence from the interior of the plateau supporting the theory of migration and interaction along the Tibetan-Yi Corridor. Although qpAdm modeling suggested that the Mbc3.5k_outlier individual could be modeled as a mixture of Basal Asian Xingyi_EN-related ancestry and Shamanka_EN,^51^ this result should be interpreted cautiously due to the lack of better proxy populations currently available to represent the eastern Eurasian ancestry component. Shamanka_EN in this case likely does not indicate direct ancestry, but serves as a proxy for a broader northern Eurasian forager ancestry, possibly linked to widespread microblade-associated hunter-gatherer groups across northern Asia.^5^^,^^51^ As a forager population uninfluenced by agriculture, Shamanka_EN may jointly with Xingyi_EN reflect the complexity of early foraging ancestry in this region, and their contributions to the Mbc3.5k_outlier may reflect remnants of earlier hunter-gatherer-related ancestries in the Tibetan Plateau during the mid-Holocene.

Secondly, we investigated the dynamic population interactions on the southern Tibetan Plateau over time. A notable observation is the admixture of lowland East Asian ancestry, including ancestry from Yellow River farmers related to pre-Shimao populations, dating to 4,764-5,100 BP. Previous studies have linked this migration to the introduction of millet farming technologies to the plateau.^4^^,^^13^ Genetic evidence indicates that Yellow River farmers appeared at sites such as Zongri (represented by Zongri4.7k populations)^20^ and dispersed across eastern plateau borders, including sites in Sichuan (Gaoshan site) and Yunnan (Haimenkou site).^52^ From our observation, the influence of Neolithic migrations from the Yellow River also reached high-altitude regions of the southern Tibetan Plateau, contributing significantly to the genetic makeup of the Mabu Co population and shaping its fine-scale genetic structure. Interestingly, unlike previously sampled ancient southern Tibetan Plateau populations,^22^ we do not observe genetic interactions between Mabu Co individuals and South or Central Asians. Although the presence of the G2 component in Mabu Co shows genetic affinity with populations from archaeological sites on the southern slopes of the Himalayas in Nepal, suggesting possible trans-Himalayan interactions around 3400 BP,^19^ it remains uncertain whether such connections extended into the broader South Asian plains. While archaeological records indicate that goods from Southwest and South Asia, as well as domesticated crops and livestock introduced from Central Asia reached the Plateau prior to 3500 BP,^29^^,^^32^^,^^33^ genetic signals of South or Central Asian ancestry have so far only been observed in individuals from the western Plateau dating to a later period, around 2300 BP,^22^ and our observation suggests such exchanges may not be widespread across the entire southern Plateau around 4400 BP. Beyond external gene flow, the genetic landscape of the Tibetan Plateau was likely shaped by complex geographic factors. The mountainous terrain may have limited human mobility and promoted genetic isolation, while major rivers such as the Yarlung Tsangpo could have served as natural corridors, enabling long-distance movement. These geographic factors, combined with limited sampling across the Plateau, may affect the resolution and accuracy of admixture models.^53^ Interpretations regarding basal ancestry and external introgression should be considered tentative, as the observed signals may reflect a broader gene-flow landscape with unsampled populations. The formation and interactions of discrete subpopulations within the Plateau also remain difficult to resolve. Particularly given the limited number of individuals available for the late period, which limits the confidence of population-level inferences, the timing, scale, and nature of these interactions require further investigation using expanded datasets.

Lastly, we also observed fine differentiation between males and females in shaping the Mabu Co population structure. We found that the diversity of maternally inherited mitochondrial genomes was higher than the paternally inherited Y chromosome. While the mitochondrial haplogroups are mostly typical of Tibetans, the Y chromosome is dominated by haplogroups typical of lowland East Asians.^54^ This suggests that lowland male farmers not only introduced agricultural techniques to the plateau but also played a significant role in shaping the genetic makeup of the Mabu Co population. Interestingly, *EPAS1*, a key allele for high-altitude adaptation, was initially observed in females, but later the males started to develop adaptive variance over time within the Mabu Co site. Given the absence of females in the late period, we restricted comparative analysis of sex-specific *EPAS1* gene distribution to the early and middle periods using Fisher’s exact test. A marginally significant *p* value (here we choose a significance threshold of 0.1, which is commonly used in small sample size tests) is obtained in early-stage gene carriage rates between sexes (two-sided *p* = 0.077), while such significance is not observed in the middle period (*p* = 1.000), suggesting a shift in the distribution of *EPAS1* allele carriers from mostly females to both males and females over time. To explore whether this pattern of sex-biased distribution for adaptive alleles existed more broadly across the plateau during its early period (>4000 BP), we compiled published EPAS1 genotype data from 127 ancient individuals (Table S13). Among the 39 individuals dating older than 4000 BP, we observed a similar sex difference in EPAS1 carriage frequency to that seen at Mabu Co: males carried the allele at a frequency of 45.8% (11/24), whereas females carried it at 73.3% (11/15). However, it is worth mentioning that the statistical power of the above analyses is limited, as only a small number of individuals are available.

Alongside these genetic shifts, cultural diversification was evident. Burial practices at the Mabu Co site became increasingly varied over time. In the early period, the adoption of extended prone burial (P), a similar burial practice in the upper Yellow River in the Qinghai region in the late Neolithic,^55^ was dominant in the majority of the burials (Figure 3), while in the middle and late periods, the presence of a variety of other burial types, such as secondary burials (S) and fetal position burials (F). Similarly, the discovery of a richer array of exotic artifacts underscores increasing cultural interaction and diversity.

In summary, paleogenomics from the southern Tibetan Plateau reconstructs the population dynamics changes at early periods of population settlement at high altitudes. While the ancient southern Tibetan Plateau populations were connected with the agricultural populations of the northeast, this southern plateau ancestry appears to have persisted since 4400 BP and contributed broadly to surrounding regions. Additionally, there was also interaction with the Basal Asian Xingyi-related ancestry along the Tibetan-Yi corridor from southwestern China. Although based on a limited number of individuals, the observed variation in maternal lineages and the slightly higher frequency of females carrying adaptive alleles may suggest differences in the demographic histories of males and females during early periods on the Plateau. Our novel scenario of plateau ancestry persists and changes in the Mabu Co site, fills in the limited understanding of the prehistoric genetic history on the southern Tibetan Plateau, and also informs the basis for the study of plateau archaeological cultures and the human behavior for adapting to the plateau.

### Limitations of the study

Our study is based on a relatively small number of individuals from one site, which limits the resolution of population-level patterns, particularly in later periods. The ancestry signals we report, such as those related to Xingyi_EN and Shamanka_EN, should be viewed as tentative given the current lack of more suitable reference populations. Geographic coverage across the Tibetan Plateau also remains incomplete, leaving some aspects of gene flow and subpopulation structure unresolved. Finally, the analyses of sex-specific processes and adaptive alleles, including EPAS1, are constrained by sample size, reducing statistical confidence. Expanded datasets in the future will help to clarify and strengthen these observations.

## Resource availability

### Lead contact

Further information and requests for resources should be directed to and will be fulfilled by the lead contact: Qiaomei Fu (fuqiaomei@ivpp.ac.cn).

### Materials availability

This study did not generate new unique reagents.

### Data and code availability

The aligned sequences are available through the Genome Sequence Archive (https://bigd.big.ac.cn/gsa-human) with the accession number: GSA: PRJCA033190, and the genotype SNP data in geno. format generated in this study has been submitted to an OMIX database (https://ngdc.cncb.ac.cn/omix), under accession number: OMIX: OMIX008140, China National Center for Bioinformation, Beijing Institute of Genomics, Chinese Academy of Sciences.^56^^,^^57^

## Acknowledgments

We are grateful to the valuable support of National Cultural Heritage Administration and Tibetan Cultural Heritage Administration. This work was supported by the Chinese Academy of Science (YSBR-019) (to Q.F.); the 10.13039/501100001809National Natural Science Foundation of China (41925009) (to Q.F.); the 10.13039/501100001809National Natural Science Foundation of China (41988101) (to F.C.); the 10.13039/501100002367Chinese Academy of Sciences (2023000065) (to Y.L.); the 10.13039/501100001809National Natural Science Foundation of China (41930323) (to X.Y.); the Second Tibetan Plateau Scientific Expedition and Research Program (2019QZKK060) (to F.C., X.Y., S.W. and Y.G.); the Youth Innovation Promotion Association of the 10.13039/501100002367Chinese Academy of Sciences (2022068) (to Y.G.).

## Author contributions

Q.F. and F.H. designed and supervised the research project. X.W., X.Y., Y.G., J.Y., S.C., J.K., N.J., Y.L., Y.T., and F.L. assembled archaeological materials and performed dating. Q.F. C.P., F.L., Q.D., R.Y., and W.P. performed or supervised wet laboratory work. X.F. and Q.F. processed the data. J.K., Q.F., T.W., Y.L., and H.S. analyzed the data. J.K., Q.F., and Y.L. wrote the article and supplements. Q.F., J.K., Y.L., and T.W. revised the article. All authors discussed, critically revised, and approved the final version of the article.

## Declaration of interests

The authors declare no competing interests.

## STAR★Methods

### Key resources table

**Table:** 

REAGENT or RESOURCE	SOURCE	IDENTIFIER
**Biological samples**		

Ancient skeletal element	This paper	See Table S1 for Specimen ID

**Chemicals, peptides, and recombinant proteins**		

Tween 20	Sigma	cat#P5927
1 M Tris-HCl, pH 8.0	AppliChem	A4577,0500
0.5 M EDTA, pH 8.0	AppliChem	A4892,1000
5 M NaCl	Sigma	S5150
25 mM each dNTP mix	Fermentas	cat#R1121
USER enzyme (mixture of Uracil-DNAglycosylase, UDG, and Endonuclease, EndoVIII)	NEB	cat#M5505L
Bst DNA Polymerase, Large Fragment	NEB	cat#M0275S
ATP, 10 mM stock solution	NEB	cat#9804
T4 Polynucleotide Kinase (10 U/mL)	NEB	cat#M0236L or S
T4 DNA Polymerase (3 U/mL)	NEB	cat#M0203L
Quick Ligation Kit	NEB	cat#M2200L
2x HI-RPM hybridization buffer	Agilent	5118–5380
20% SDS	Serva	39575.01
SSC buffer	Ambion	AM9770
AmpliTaq Gold 10x PCR buffer without Mg	Life Technologies	4379874
1M NaOH	Sigma	71463-1L
3M Sodium acetate pH 5.2	Sigma	S7899
Dynabeads MyOne C1	Life Technologies	65002
SeraMag Speedbeads	GE	65152105050250
Cot-1 DNA	Invitrogen	15279011

**Deposited data**		

The raw sequence reads and aligned BAM files reported in this paper have been deposited in the Genome Sequence Archive (Genomics, Proteomics & Bioinformatics 2021) in National Genomics Data Center (Nuclear Acids Res 2022), China National Center for Bioinformation/Beijing Institute of Genomics, Chinese Academy of Sciences (https://ngdc.cncb.ac.cn/gsa-human)	This paper	GSA: PRJCA033190
The pseudo-diploid genotype calls (Eigenstrat format) data reported in this paper have been deposited in the OMIX, China National Center for Bioinformation/Beijing Institute of Genomics, Chinese Academy of Sciences (https://ngdc.cncb.ac.cn/omix)	This paper	OMIX: OMIX008140

**Oligonucleotides**		

Probe for 1240K Panel	Supplemental Data 2a of Haak et al.^58^	https://static-content.springer.com/esm/art%3A10.1038%2Fnature14317/MediaObjects/41586_2015_ BFnature14317_MOESM31_ESM.zip
Probe for 1240K Panel	Supplemental Data 2b of Haak et al.^58^	https://static-content.springer.com/esm/art%3A10.1038%2Fnature14317/MediaObjects/41586_2015_ BFnature14317_MOESM31_ESM.zip
Probe for 1240K Panel	Supplemental Data 2c of Haak et al.^58^	https://static-content.springer.com/esm/art%3A10.1038%2Fnature14317/MediaObjects/41586_2015_ BFnature14317_MOESM31_ESM.zip
Probe for 1240K Panel	Supplemental Data 2d of Haak et al.^58^	https://static-content.springer.com/esm/art%3A10.1038%2Fnature14317/MediaObjects/41586_2015_ BFnature14317_MOESM31_ESM.zip
Probe for 1240K Panel	Supplemental Data 1a of Fu et al.^59^	https://static-content.springer.com/esm/art%3A10.1038%2Fnature14558/MediaObjects/41586_2015_BFnature14558_MOESM242_ESM.zip
Probe for 1240K Panel	Supplemental Data 1b of Fu et al.^59^	https://static-content.springer.com/esm/art%3A10.1038%2Fnature14558/MediaObjects/41586_2015_BFnature14558_MOESM242_ESM.zip
Probe for 1240K Panel	Supplemental Data 1c of Fu et al.^59^	https://static-content.springer.com/esm/art%3A10.1038%2Fnature14558/MediaObjects/41586_2015_BFnature14558_MOESM242_ESM.zip
Phosphate-AGATCGGAAG[C3Spacer]10 [TEG-biotin] (TEG = triethylene glycol spacer)	Gansauge and Meyer^60^	CL78 Single-stranded adaptor
GTGACTGGAGTTCAGACGTT GCTCTTCC∗GA∗TC∗T	Gansauge and Meyer^60^	CL130 Extension primer
A^∗^C^∗^A^∗^C^∗^TCTTTCCCTACACGAC GCTCTTCCGATCT^∗^G^∗^T^∗^C^∗^T	Meyer and Kircher^61^	NI7_P5_CR2_short
G^∗^T^∗^G^∗^A^∗^CTGGAGTTCAGACGTGTG CTCTTCCGATCT^∗^G^∗^T^∗^C^∗^T	Meyer and Kircher^61^	NI8_P7_CR2_short
A^∗^G^∗^A^∗^C^∗^AGATC^∗^G^∗^G^∗^A^∗^A	Meyer and Kircher^61^	NI9_P5P7_CR2_comp
GTGACTGGAGTTCAGACG TGTGCTCTTCCGATCT-Phosphate	Fu et al.^59^^,^^62^	BO4.P7.part1.R
CAAGCAGAAGACGGCAT ACGAGAT-Phosphate	Fu et al.^59^^,^^62^	BO6.P7.part2.R
GTGTAGATCTCGGTGGTC GCCGTATCATT-Phosphate	Fu et al.^59^^,^^62^	BO8.P5.part1.R
AGATCGGAAGAGCGTC GTGTAGGGAAAGAGTGT-Phosphate	Fu et al.^59^^,^^62^	BO10.P5.part2.R
GGAAGAGCGTCGTGTAGG GAAAGAGTGT-Phosphate	Yang et al.^63^	BO11.P5.part2.R

**Software and algorithms**		

leeHom	Renaud et al.^64^	https://github.com/grenaud/leeHom/; RRID: SCR_002710
BWA 0.7.17	Li and Durbin^65^	http://bio-bwa.sourceforge.net/; RRID: SCR_010910
ContamMix 1.0–10	Fu et al.^66^	https://github.com/StanfordBioinformatics/DEFUNCT-env-modules/tree/master/contamMix/
ANGSD 0.921	Komeliussen et al.^67^	http://popgen.dk/angsd/index.php/ANGSD/
READ	Monroy Kuhn et al.^68^	https://bitbucket.org/tguenther/read/src/master/
Yleaf 2.2	Ralf et al.^69^	https://github.com/genid/Yleaf
EIGENSOFT 6.1.4	Patterson et al.^70^	https://github.com/DReichLab/EIG/; RRID: SCR_004965
ADMIXTURE 1.3.0	Alexander et al.^71^	http://dalexander.github.io/admixture/download.html; RRID: SCR_001263
PLINK v1.9	Purcell et al.^72^	https://www.cog-genomics.org/plink/1.9; RRID: SCR_001757
ADMIXTOOLS (qp3Pop, qpDstat, qpAdm, qpGraph)	Patterson et al.^73^	https://github.com/DReichLab/AdmixTools/; RRID:SCR_018495
DATES_v4010	Chintalapati et al.^74^	https://github.com/MoorjaniLab/DATES_v4010
Samtools v1.5	Danecek et al.^75^	https://github.com/samtools/samtools; RRID:SCR_002105
Treemix v1.13	Pickrell and Pritchard^76^	https://bitbucket.org/nygcresearch/treemix
GLIMPSE v2.0.0	Rubinacci et al.^77^	https://github.com/odelaneau/GLIMPSE
RELATE v1.2.1	Speidel et al.^78^^,^^79^	https://myersgroup.github.io/relate/

### Method details

#### Ancient human bone sampling

Samples of human remains were collected during the 2021, 2022 and 2023 Mabu Co archaeological excavations. Using sterile scalpels and knives under biochemical protective clothing, we sampled the petrous bone and tooth from the human bone into sterile sample bags, which were then sealed and brought to the ancient DNA wet laboratory at the Institute of Vertebrate Paleontology and Paleoanthropology, Chinese Academy of Sciences, in Beijing, China.

#### DNA extraction and in solution capture

For all 16 samples DNA was extracted from less than 100 mg of bone powder using an optimized silica-based extraction protocol.^80^ Single-stranded DNA libraries were prepared using standard single-stranded (SS) protocols.^60^ For the two new samples from the 2021 excavation, the libraries were built without UDG treatment, and all other samples were subjected to uracil-DNA-glycosylase treatment^81^ for reducing the ancient DNA deaminated bases at the end of the strand (Table S1). For the 16 successful library samples, solution hybridization oligonucleotide probes were used to enrich endogenous DNA for a complete mitochondrial genome^62^ and 1.2 million nuclear single nucleotide polymorphisms (“1240k” SNPs).^58^^,^^59^

#### Sequencing and reads alignment

Mitochondrial DNA libraries were sequenced on Illumina HiSeq XTen machines (2 × 76 bp reads), and nuclear DNA libraries on Illumina HiSeq 2500 machines (2 × 150 bp reads). Adaptor trimming and merging of sequences with a minimum overlap of 11 base pairs were conducted using leeHom.^64^ Merged reads of at least 30 bp were aligned to the revised Cambridge Reference Sequence (rCRS) for mitochondrial DNA and hg19 for nuclear DNA using BWA^65^ (v0.7.17). Duplicate reads with identical orientation, start, and end positions were removed, as were fragments with mapping quality scores below 30.

#### Test for contamination and genotyping

Ancient DNA authenticity was assessed by identifying C-T deamination damage signatures. Contemporary human contamination rates were estimated using ContamMix,^66^ with all samples exhibiting low contamination levels (0–1.6%). Pseudo-haploid genotypes were determined by randomly sampling one fragment per position. For UDG-treated samples, 2 bp read ends were masked; for non-UDG-treated samples, 5 bp ends were masked.

#### Determination of Uniparental haplogroups

Mitochondrial haplogroups were assigned to each individual using Haplogrep2,^82^ referencing Phylotree Build 17.^83^ For males, Y-haplogroups were determined with Yleaf (v2.2),^69^ considering only sequences with mapping and base quality scores of 30 or higher.

#### Dataset for population analysis

Mabu Co populations were analyzed using a worldwide genetic dataset encompassing 35 present-day populations from the Human Origins (HO) SNP panel,^73^ Simons Genome Diversity Panel (SGDP),^84^ Human Genome Diversity Project (HGDP),^85^ and Tibetan-related publications.^20^

#### Kinship analysis

Genotyped data for 16 new individuals were combined with 9 previously published individuals, yielding a dataset of 25 individuals. Data were converted to TPED format using PLINK (v1.9)^72^ with parameters “—allele ACGT –recode transpose,” and kinship was inferred via average pairwise P0 values calculated using READ software.^68^ To avoid confounding effects of relatedness, we excluded kinship pairs from downstream population genetic analyses. Our exclusion protocol for kinship individuals prioritized retaining: 1) directly radiocarbon-dated individuals when both relatives met data quality thresholds; 2) the individual with higher coverage depth when both had radiocarbon dates. Retained status of each individual is annotated in Table S1.

#### Principal components analysis

PCA was performed with smartpca (v16000) in EIGENSOFT (v7.2.1)^70^ using parameters: numoutlierevec: 2, outliersigmathresh: 12, lsqproject: YES, autoshrink: YES. Ancient individuals were projected onto the principal component space constructed by present-day East Asian populations and near the Tibetan Plateau genotyped in the Human Origins dataset.

#### ADMIXTURE

ADMIXTURE is a method for fast model-based estimation of ancestry in unrelated individuals.^71^ Linkage disequilibrium pruning was conducted using PLINK (v1.9) with parameters “--indep-pairwise 200 25 0.4.” We then used ADMIXTURE (v1.30) to estimate individual ancestries and determine population structure parameters “-j10,” running the software 10 times using different seeds to estimate the lowest CV (cross validation) error for the ‘best’ K. Results were plotted using AdmixturePlotter-master R script.

#### Outgroup f3 statistics

The outgroup f3 statistics quantified genetic drift and tested admixture using qp3Pop (v412) from ADMIXTOOLS.^73^ The Mbuti population served as the outgroup in the form f3(Out; X, Y). Higher f3 values indicated genetic similarity, and results were visualized via heatmaps.

#### f4 statistics

The f4 statistics were calculated with qpDstat (v712)^73^ using the parameter f4mode: YES. Autosomal SNPs from the 1240k dataset were analyzed in the form f4(Mbuti, P2; P3, P4), where Z-scores indicated allele-sharing patterns.

#### qpAdm analysis

qpAdm is a method based on the f4-statistic that models the mixture of ancestry for a target population.^73^ We used qpAdm (v634) in the ADMIXTOOLS package with the parameters “details: YES” and “allsnps: YES” to model ancient Mabu Co populations using genetic libraries from both ancient Tibetans and ancient lowland East Asian populations. In detail, qpAdm rotating strategy were used as Right populations including “Mbuti, UstIshim, Yana, Ganj_Dareh_N, Kostenki14,” Left populations contain two panels, the first panel used as rotating panel (populations were listed as potential ancestry source, if not used as source for target population, then will be listed into Right populations) including “Tianyuan, Onge, Xingyi_EN, Fujian_EN, AR14-10K, Shandong_EN, preShimao, Yumin,” the second panel used as no rotating including “Mbc4.4k_G0, Mbc4.4k_G1, Mbc4.4k_G2, Zongri5.1k, Shamanka_EN, YR_MN_WGM” for modeling middle and late periods populations by using early Mabu Co populations as a potential ancestral component.

#### TreeMix

We inferred maximum-likelihood population admixture graphs using TreeMix (v1.13).^76^ The trees were rooted with the Mbuti population, and a block size of 1000 SNPs was specified (“-k 1000”). To assess the support for tree topology, we performed 1000 bootstrap replicates (“-bootstrap -q”).

#### Admixture time inference by DATES

DATES (Distribution of Ancestry Tracts of Evolutionary Signals) is an admixture LD-based methods by measuring the extent of the allelic correlation across markers to infer the time of admixture.^86^ We used DATES_v4010^68^ to further investigate the admixture time between ancient south plateau ancestry and the Yellow River related populations and the Yunnan Xingyi_EN populations with default parameters “zdipcorrmode: YES, runfit: YES and afffit: YES,” and generation time of 28 years were used to convert generations of admixture into admixture time point in BP units.

#### EPAS1 haplotype

EPAS1 gene variants are identified by 30 SNPs across a ∼31.7-kb haplotype block on chromosome 2 containing Denisovan-derived introgressed variants. EPAS1 haplotype frequency was assessed using 20 highly differentiated SNPs obtained in our ancient genomic data. SNP data were extracted with Samtools (v1.5)^75^ mpileup and co-analyzed with published ancient plateau genome data in Table S13.

#### Genealogy-based chromosome painting

We applied genotype imputation to the Mabu Co data prior to RELATE analysis. Imputation was performed using GLIMPSE2^77^ with default parameters, with the high-coverage 1000 Genomes dataset (lifted over from hg19) serving as the reference panel. The resulting imputed data were then merged with datasets from Xingyi, ancient Tibetans, and northern East Asians that had been imputed using the same procedure.^23^ Variants were filtered to retain SNPs with minor allele frequency (MAF) > 0.01 and genotype probability (GP) ≥ 0.8. RELATE (v1.2.1)^78^^,^^79^ was subsequently employed to infer whole-genome genealogies, assuming a per-base, per-generation mutation rate of 4 × 10^−9^ and using pre-inferred average coalescence rates from the Simons Genome Diversity Project (SGDP). Finally, we constructed ancestry models based on a genealogy-based chromosome painting framework^23^ to assess the presence of ghost ancestry in ancient Tibetan population.

## Supplemental information

Document S1. Figures S1–S12

Table S1. Samples information summary, including library, snp coverage, contamination, mtDNA haplogroup, Y-chr haplogroup and dating, related to Figures 1 and 3

Table S2. Raw data for individual pairwise f4 heatmap plotting with significant differences (|Z| > 3) assessing genetic affinities among the Mabu Co individuals, related to Figure 4

Table S3. F4-statistics showing the genetic connections between Mbc4.4k_G0 and Zongri5.1k, related to Figure 4

Table S4. QpAdm models of Mabu Co Early period groups and Zongri5.1k population, related to Figure 5

Table S5. F4-statistics show the genetic differences between Mbc4.4k_G0 and Mbc4.4k_G1, and between Mbc4.4k_G0 and Mbc4.4k_G2, related to Figure 4

Table S6. DATES results inferring admixture time of Mbc4.4k_G1, Mbc4.4k_G2 and Mbc3.5k_G0, related to Figure 5

Table S7. F4-statistics show the genetic continuity among Mbc4.4k_G2, Mbc4k_G2, Shannan3k, Lubrak, related to Figure 4

Table S8. QpAdm models of Mabu Co Middle/Late period groups and Shannan3k/Lubrak populations, related to Figure 5

Table S9. F4-statistics [F4(Mbc3.5k_outlier, col.; row., Mbuti)] showing close genetic connections of Mbc3.5k_outlier with Xingyi_EN, related to Figure 5

Table S10. DATES results for Mbc3.5k_outlier admixture time, related to Figure 5

Table S11. mtDNA haplogroups with detailed mutation information, related to Figure 3

Table S12. EPAS1 allele summary in Mabu Co individuals, related to Figure 3

Table S13. EPAS1 Genotype information from the Mabu Co and previously published ancient Tibetan genomes, related to Figure 3

Table S14. Sites information of previously published paleogenomic data on the Tibetan Plateau, related to Figure 1

## References

[1] Chen F., Welker F., Shen C.C., Bailey S.E., Bergmann I., Davis S., Xia H., Wang H., Fischer R., Freidline S.E. (2019). A late Middle Pleistocene Denisovan mandible from the Tibetan Plateau. Nature.

[2] Zhang D., Xia H., Chen F., Li B., Slon V., Cheng T., Yang R., Jacobs Z., Dai Q., Massilani D. (2020). Denisovan DNA in Late Pleistocene sediments from Baishiya Karst Cave on the Tibetan Plateau. Science.

[3] Xia H., Zhang D., Wang J., Fagernäs Z., Li T., Li Y., Yao J., Lin D., Troché G., Smith G.M. (2024). Middle and Late Pleistocene Denisovan subsistence at Baishiya Karst Cave. Nature.

[4] d’Alpoim Guedes J., Aldenderfer M. (2019). The Archaeology of the Early Tibetan Plateau: New Research on the Initial Peopling through the Early Bronze Age. J. Archaeol. Res..

[5] Zhang X., Jin Y., He W., Yi M., Xu X. (2020). A consideration of the spatiotemporal distribution of microblade industries on the Tibetan Plateau. Quat. Int..

[6] Lu D., Lou H., Yuan K., Wang X., Wang Y., Zhang C., Lu Y., Yang X., Deng L., Zhou Y. (2016). Ancestral Origins and Genetic History of Tibetan Highlanders. Am. J. Hum. Genet..

[7] Li Y.C., Tian J.Y., Liu F.W., Yang B.Y., Gu K.S.Y., Rahman Z.U., Yang L.Q., Chen F.H., Dong G.H., Kong Q.P. (2019). Neolithic millet farmers contributed to the permanent settlement of the Tibetan Plateau by adopting barley agriculture. Natl. Sci. Rev..

[8] Sagart L., Jacques G., Lai Y., Ryder R.J., Thouzeau V., Greenhill S.J., List J.M. (2019). Dated language phylogenies shed light on the ancestry of Sino-Tibetan. Proc. Natl. Acad. Sci. USA.

[9] Zhang M., Yan S., Pan W., Jin L. (2019). Phylogenetic evidence for Sino-Tibetan origin in northern China in the Late Neolithic. Nature.

[10] Yang J., Gao Y., Wang Y., Chen S., Ran J., Yang X. (2024). The spread of ancient crops through the “Plateau Road”. Quat. Sci..

[11] Yang X., Gao Y., Wangdue S., Ran J., Wang Q., Chen S., Yang J., Wang T., Gu Z., Zhang Y. (2024). Lake-centred sedentary lifestyle of early Tibetan Plateau Indigenous populations at high elevation 4,400 years ago. Nat. Ecol. Evol..

[12] Wang Q., Zhang Y., Chen S., Gao Y., Yang J., Ran J., Gu Z., Yang X. (2023). Human sedentism and use of animal resources on the prehistoric Tibetan Plateau. J. Geogr. Sci..

[13] Chen F.H., Dong G.H., Zhang D.J., Liu X.Y., Jia X., An C.B., Ma M.M., Xie Y.W., Barton L., Ren X.Y. (2015). Agriculture facilitated permanent human occupation of the Tibetan Plateau after 3600 BP. Science.

[14] Gao Y., Yang J., Ma Z., Tong Y., Yang X. (2021). New evidence from the Qugong site in the central Tibetan Plateau for the prehistoric Highland Silk Road. Holocene.

[15] Huo W. (2014). A Preliminary Study on the Early Metal Objects and the Early Metal Age in Tibet. Acta Archaeol. Sin..

[16] Huo W. (2024). Insights into the Early Metal Age on the Tibetan Plateau from the Discovery of the “Yak Mirror” in Tibet. China Tibetol..

[17] Jeong C., Ozga A.T., Witonsky D.B., Malmström H., Edlund H., Hofman C.A., Hagan R.W., Jakobsson M., Lewis C.M., Aldenderfer M.S. (2016). Long-term genetic stability and a high-altitude East Asian origin for the peoples of the high valleys of the Himalayan arc. Proc. Natl. Acad. Sci. USA.

[18] Ding M., Wang T., Ko A.M.S., Chen H., Wang H., Dong G., Lu H., He W., Wangdue S., Yuan H. (2020). Ancient mitogenomes show plateau populations from last 5200 years partially contributed to present-day Tibetans. Proc. Biol. Sci..

[19] Liu C.C., Witonsky D., Gosling A., Lee J.H., Ringbauer H., Hagan R., Patel N., Stahl R., Novembre J., Aldenderfer M. (2022). Ancient genomes from the Himalayas illuminate the genetic history of Tibetans and their Tibeto-Burman speaking neighbors. Nat. Commun..

[20] Wang H., Yang M.A., Wangdue S., Lu H., Chen H., Li L., Dong G., Tsring T., Yuan H., He W. (2023). Human genetic history on the Tibetan Plateau in the past 5100 years. Sci. Adv..

[21] Zhang G., Cui C., Wangdue S., Lu H., Chen H., Xi L., He W., Yuan H., Tsring T., Chen Z. (2023). Maternal genetic history of ancient Tibetans over the past 4000 years. J. Genet. Genomics.

[22] Bai F., Liu Y., Wangdue S., Wang T., He W., Xi L., Tsho Y., Tsering T., Cao P., Dai Q. (2024). Ancient genomes revealed the complex human interactions of the ancient western Tibetans. Curr. Biol..

[23] Wang T., Yang M.A., Zhu Z., Ma M., Shi H., Speidel L., Min R., Yuan H., Jiang Z., Hu C. (2025). Prehistoric genomes from Yunnan reveal ancestry related to Tibetans and Austroasiatic speakers. Science.

[24] Chen F., Zhang J., Liu J., Cao X., Hou J., Zhu L., Xu X., Liu X., Wang M., Wu D. (2020). Climate change, vegetation history, and landscape responses on the Tibetan Plateau during the Holocene: A comprehensive review. Quat. Sci. Rev..

[25] Zhao M., Kong Q.P., Wang H.W., Peng M.S., Xie X.D., Wang W.Z., Zhao S.N., Jiayang, Duan J.G., Cai M.C. (2009). Mitochondrial genome evidence reveals successful Late Paleolithic settlement on the Tibetan Plateau. Proc. Natl. Acad. Sci. USA.

[26] Qi X., Cui C., Peng Y., Zhang X., Yang Z., Zhong H., Zhang H., Xiang K., Cao X., Wang Y. (2013). Genetic evidence of paleolithic colonization and neolithic expansion of modern humans on the tibetan plateau. Mol. Biol. Evol..

[27] Liu L., Chen J., Wang J., Zhao Y., Chen X. (2022). Archaeological evidence for initial migration of Neolithic Proto Sino-Tibetan speakers from Yellow River valley to Tibetan Plateau. Proc. Natl. Acad. Sci. USA.

[28] Institute of Cultural Relics Protection of Tibet Autonomous Region, S.A.I., Zhada Bureau of Cultural Heritage (2022).

[29] Cao S., Wen R., Yu C., Shargan W., Tash T., Wang D. (2021). New evidence of long-distance interaction across the Himalayas: Faience beads from Western Tibet. J. Cult. Herit..

[30] Lu H., Chen X., Zhang Z., Tang L., Lemoine X., Wangdue S., Chen Z., Liu X., Frachetti M.D. (2021). Early agropastoral settlement and cultural change in central Tibet in the first millennium BC: excavations at Bangga. Antiquity.

[31] Qiu Q., Wang L., Wang K., Yang Y., Ma T., Wang Z., Zhang X., Ni Z., Hou F., Long R. (2015). Yak whole-genome resequencing reveals domestication signatures and prehistoric population expansions. Nat. Commun..

[32] Huo W. (2023).

[33] Tong T. (2022).

[34] Mischke S., Weynell M., Zhang C.J., Wiechert U. (2013). Spatial variability of 14C reservoir effects in Tibetan Plateau lakes. Quat. Int..

[35] Peng M.-S., Palanichamy M.G., Yao Y.-G., Mitra B., Cheng Y.-T., Zhao M., Liu J., Wang H.-W., Pan H., Wang W.-Z. (2011). Inland post-glacial dispersal in East Asia revealed by mitochondrial haplogroup M9a'b. BMC Biol..

[36] Derenko M., Malyarchuk B., Denisova G., Perkova M., Rogalla U., Grzybowski T., Khusnutdinova E., Dambueva I., Zakharov I. (2012). Complete Mitochondrial DNA Analysis of Eastern Eurasian Haplogroups Rarely Found in Populations of Northern Asia and Eastern Europe. PLoS One.

[37] Askapuli A., Vilar M., Garcia-Ortiz H., Zhabagin M., Sabitov Z., Akilzhanova A., Ramanculov E., Schamiloglu U., Martinez-Hernandez A., Contreras-Cubas C. (2022). Kazak mitochondrial genomes provide insights into the human population history of Central Eurasia. PLoS One.

[38] Li X., Zhang X., Yu T., Ye L., Huang T., Chen Y., Liu S., Wen Y. (2023). Whole mitochondrial genome analysis in highland Tibetans: further matrilineal genetic structure exploration. Front. Genet..

[39] Kutanan W., Kampuansai J., Brunelli A., Ghirotto S., Pittayaporn P., Ruangchai S., Schröder R., Macholdt E., Srikummool M., Kangwanpong D. (2018). New insights from Thailand into the maternal genetic history of Mainland Southeast Asia. Eur. J. Hum. Genet..

[40] Wang C.-C., Wang L.-X., Shrestha R., Zhang M., Huang X.-Y., Hu K., Jin L., Li H. (2014). Genetic Structure of Qiangic Populations Residing in the Western Sichuan Corridor. PLoS One.

[41] Yao X., Tang S., Bian B., Wu X., Chen G., Wang C.-C. (2017). Improved phylogenetic resolution for Y-chromosome Haplogroup O2a1c-002611. Sci. Rep..

[42] Wang M., Wang Z., He G., Wang S., Zou X., Liu J., Wang F., Ye Z., Hou Y. (2020). Whole mitochondrial genome analysis of highland Tibetan ethnicity using massively parallel sequencing. Forensic Sci. Int. Genet..

[43] Wang M., Huang Y., Liu K., Wang Z., Zhang M., Yuan H., Duan S., Wei L., Yao H., Sun Q. (2024). Multiple human population movements and cultural dispersal events shaped the landscape of Chinese paternal heritage. Mol. Biol. Evol..

[44] Fan G.-Y., Song D.-L., Jin H.-Y., Zheng X.-K. (2021). Gene flow and phylogenetic analyses of paternal lineages in the Yi-Luo valley using Y-STR genetic markers. Ann. Hum. Biol..

[45] Yi X., Liang Y., Huerta-Sanchez E., Jin X., Cuo Z.X.P., Pool J.E., Xu X., Jiang H., Vinckenbosch N., Korneliussen T.S. (2010). Sequencing of 50 human exomes reveals adaptation to high altitude. Science.

[46] Huerta-Sanchez E., Jin X., Asan, Bianba Z., Peter B.M., Vinckenbosch N., Liang Y., Yi X., He M., Somel M. (2014). Altitude adaptation in Tibetans caused by introgression of Denisovan-like DNA. Nature.

[47] Zhang X., Witt K.E., Bañuelos M.M., Ko A., Yuan K., Xu S., Nielsen R., Huerta-Sanchez E. (2021). The history and evolution of the Denisovan-EPAS1 haplotype in Tibetans. Proc. Natl. Acad. Sci. USA.

[48] Shi S. (2018). Ethnic flows in the Tibetan-Yi corridor throughout history. Int. J. Anthropol. Ethnol..

[49] Yang Z., Jin Y., Tan Y., Ge J., Wang S., Gao X., Olsen J.W., Zhang X. (2024). Terminal Pleistocene Human Occupation of the Qomolangma Region: New Evidence from the Su-re Site. Land.

[50] Ma M., Lu Y., Dong G., Ren L., Min R., Kang L., Zhu Z., Li X., Li B., Yang Z. (2022). Understanding the transport networks complex between South Asia, Southeast Asia and China during the late Neolithic and Bronze Age. Holocene.

[51] Damgaard P.D., Martiniano R., Kamm J., Moreno-Mayar J.V., Kroonen G., Peyrot M., Barjamovic G., Rasmussen S., Zacho C., Baimukhanov N. (2018). The first horse herders and the impact of early Bronze Age steppe expansions into Asia. Science.

[52] Tao L., Yuan H., Zhu K., Liu X., Guo J., Min R., He H., Cao D., Yang X., Zhou Z. (2023). Ancient genomes reveal millet farming-related demic diffusion from the Yellow River into southwest China. Curr. Biol..

[53] Flegontova O., Işıldak U., Yüncü E., Williams M.P., Huber C.D., Kočí J., Vyazov L.A., Changmai P., Flegontov P. (2025). Performance of qpAdm-based screens for genetic admixture on graph–shaped histories and stepping stone landscapes. Genetics.

[54] Wang L.X., Lu Y., Zhang C., Wei L.H., Yan S., Huang Y.Z., Wang C.C., Mallick S., Wen S.Q., Jin L. (2018). Reconstruction of Y-chromosome phylogeny reveals two neolithic expansions of Tibeto-Burman populations. Mol. Genet. Genomics..

[55] Yuan X., Li X. (2024). Research on Prone Burial in Prehistoric China. Jianghan Archaeol..

[56] CNCB-NGDC Members and Partners (2024). Database Resources of the National Genomics Data Center, China National Center for Bioinformation in 2024. Nucleic Acids Res..

[57] Chen T., Chen X., Zhang S., Zhu J., Tang B., Wang A., Dong L., Zhang Z., Yu C., Sun Y. (2021). The Genome Sequence Archive Family: Toward Explosive Data Growth and Diverse Data Types. Genom. Proteom. Bioinform..

[58] Haak W., Lazaridis I., Patterson N., Rohland N., Mallick S., Llamas B., Brandt G., Nordenfelt S., Harney E., Stewardson K. (2015). Massive migration from the steppe was a source for Indo-European languages in Europe. Nature.

[59] Fu Q., Hajdinjak M., Moldovan O.T., Constantin S., Mallick S., Skoglund P., Patterson N., Rohland N., Lazaridis I., Nickel B. (2015). An early modern human from Romania with a recent Neanderthal ancestor. Nature.

[60] Gansauge M.T., Meyer M. (2013). Single-stranded DNA library preparation for the sequencing of ancient or damaged DNA. Nat. Protoc..

[61] Meyer M., Kircher M. (2010). Illumina sequencing library preparation for highly multiplexed target capture and sequencing. Cold Spring Harb. Protoc..

[62] Fu Q., Meyer M., Gao X., Stenzel U., Burbano H.A., Kelso J., Pääbo S. (2013). DNA analysis of an early modern human from Tianyuan Cave, China. Proc. Natl. Acad. Sci. USA.

[63] Yang M.A., Gao X., Theunert C., Tong H., Aximu-Petri A., Nickel B., Slatkin M., Meyer M., Pääbo S., Kelso J., Fu Q. (2017). 40,000-Year-Old Individual from Asia Provides Insight into Early Population Structure in Eurasia. Curr. Biol..

[64] Renaud G., Stenzel U., Kelso J. (2014). leeHom: adaptor trimming and merging for Illumina sequencing reads. Nucleic Acids Res..

[65] Li H., Durbin R. (2009). Fast and accurate short read alignment with Burrows-Wheeler transform. Bioinformatics.

[66] Fu Q., Mittnik A., Johnson P.L.F., Bos K., Lari M., Bollongino R., Sun C., Giemsch L., Schmitz R., Burger J. (2013). A Revised Timescale for Human Evolution Based on Ancient Mitochondrial Genomes. Curr. Biol..

[67] Korneliussen T.S., Albrechtsen A., Nielsen R. (2014). ANGSD: Analysis of Next Generation Sequencing Data. BMC Bioinf..

[68] Monroy Kuhn J.M., Jakobsson M., Günther T. (2018). Estimating genetic kin relationships in prehistoric populations. PLoS One.

[69] Ralf A., Montiel González D., Zhong K., Kayser M. (2018). Yleaf: Software for Human Y-Chromosomal Haplogroup Inference from Next-Generation Sequencing Data. Mol. Biol. Evol..

[70] Patterson N., Price A.L., Reich D. (2006). Population structure and eigenanalysis. PLoS Genet..

[71] Alexander D.H., Novembre J., Lange K. (2009). Fast model-based estimation of ancestry in unrelated individuals. Genome Res..

[72] Purcell S., Neale B., Todd-Brown K., Thomas L., Ferreira M.A.R., Bender D., Maller J., Sklar P., de Bakker P.I.W., Daly M.J., Sham P.C. (2007). PLINK: a tool set for whole-genome association and population-based linkage analyses. Am. J. Hum. Genet..

[73] Patterson N., Moorjani P., Luo Y., Mallick S., Rohland N., Zhan Y., Genschoreck T., Webster T., Reich D. (2012). Ancient admixture in human history. Genetics.

[74] Chintalapati M., Patterson N., Moorjani P. (2022). The spatiotemporal patterns of major human admixture events during the European Holocene. eLife.

[75] Danecek P., Bonfield J.K., Liddle J., Marshall J., Ohan V., Pollard M.O., Whitwham A., Keane T., McCarthy S.A., Davies R.M., Li H. (2021). Twelve years of SAMtools and BCFtools. GigaScience.

[76] Pickrell J.K., Pritchard J.K. (2012). Inference of Population Splits and Mixtures from Genome-Wide Allele Frequency Data. PLoS Genet..

[77] Rubinacci S., Hofmeister R.J., Sousa da Mota B., Delaneau O. (2023). Imputation of low-coverage sequencing data from 150,119 UK Biobank genomes. Nat. Genet..

[78] Speidel L., Forest M., Shi S., Myers S.R. (2019). A method for genome-wide genealogy estimation for thousands of samples. Nat. Genet..

[79] Speidel L., Cassidy L., Davies R.W., Hellenthal G., Skoglund P., Myers S.R. (2021). Inferring Population Histories for Ancient Genomes Using Genome-Wide Genealogies. Mol. Biol. Evol..

[80] Dabney J., Knapp M., Glocke I., Gansauge M.T., Weihmann A., Nickel B., Valdiosera C., García N., Pääbo S., Arsuaga J.L., Meyer M. (2013). Complete mitochondrial genome sequence of a Middle Pleistocene cave bear reconstructed from ultrashort DNA fragments. Proc. Natl. Acad. Sci. USA.

[81] Rohland N., Harney E., Mallick S., Nordenfelt S., Reich D. (2015). Partial uracil - DNA - glycosylase treatment for screening of ancient DNA. Philos. Trans. R. Soc. Lond. B Biol. Sci..

[82] Weissensteiner H., Pacher D., Kloss-Brandstätter A., Forer L., Specht G., Bandelt H.-J., Kronenberg F., Salas A., Schönherr S. (2016). HaploGrep 2: mitochondrial haplogroup classification in the era of high-throughput sequencing. Nucleic Acids Res..

[83] Van Oven M., Kayser M. (2009). Updated comprehensive phylogenetic tree of global human mitochondrial DNA variation. Hum. Mutat..

[84] Mallick S., Li H., Lipson M., Mathieson I., Gymrek M., Racimo F., Zhao M., Chennagiri N., Nordenfelt S., Tandon A. (2016). The Simons Genome Diversity Project: 300 genomes from 142 diverse populations. Nature.

[85] Bergström A., McCarthy S.A., Hui R., Almarri M.A., Ayub Q., Danecek P., Chen Y., Felkel S., Hallast P., Kamm J. (2020). Insights into human genetic variation and population history from 929 diverse genomes. Science.

[86] Moorjani P., Sankararaman S., Fu Q., Przeworski M., Patterson N., Reich D. (2016). A genetic method for dating ancient genomes provides a direct estimate of human generation interval in the last 45,000 years. Proc. Natl. Acad. Sci. USA.

