# Supplementary material for: Ancient genomes reveal basal Asian ancestries and dynamic population interactions over time on the southern Tibetan Plateau
Source: iScience. 2025 Oct 3;28(11):113676. doi: 10.1016/j.isci.2025.113676 (PMC12589885; doi:10.1016/j.isci.2025.113676)
Supplement: Document S1. Figures S1–S12 [file mmc1.pdf]

iScience, Volume *28*

Supplemental information

Ancient genomes reveal basal Asian ancestries and dynamic population interactions over time on the southern Tibetan Plateau

Jingkun Ran, Yichen Liu, Shargan Wangdue, Xiaoyan Yang, Tianyi Wang, Yu Gao, Peng Cao, Yan Tong, Qingyan Dai, Songtao Chen, Han Shi, Feng Liu, Xiaotian Feng, Yazhong Li, Fahu Chen, and Qiaomei Fu

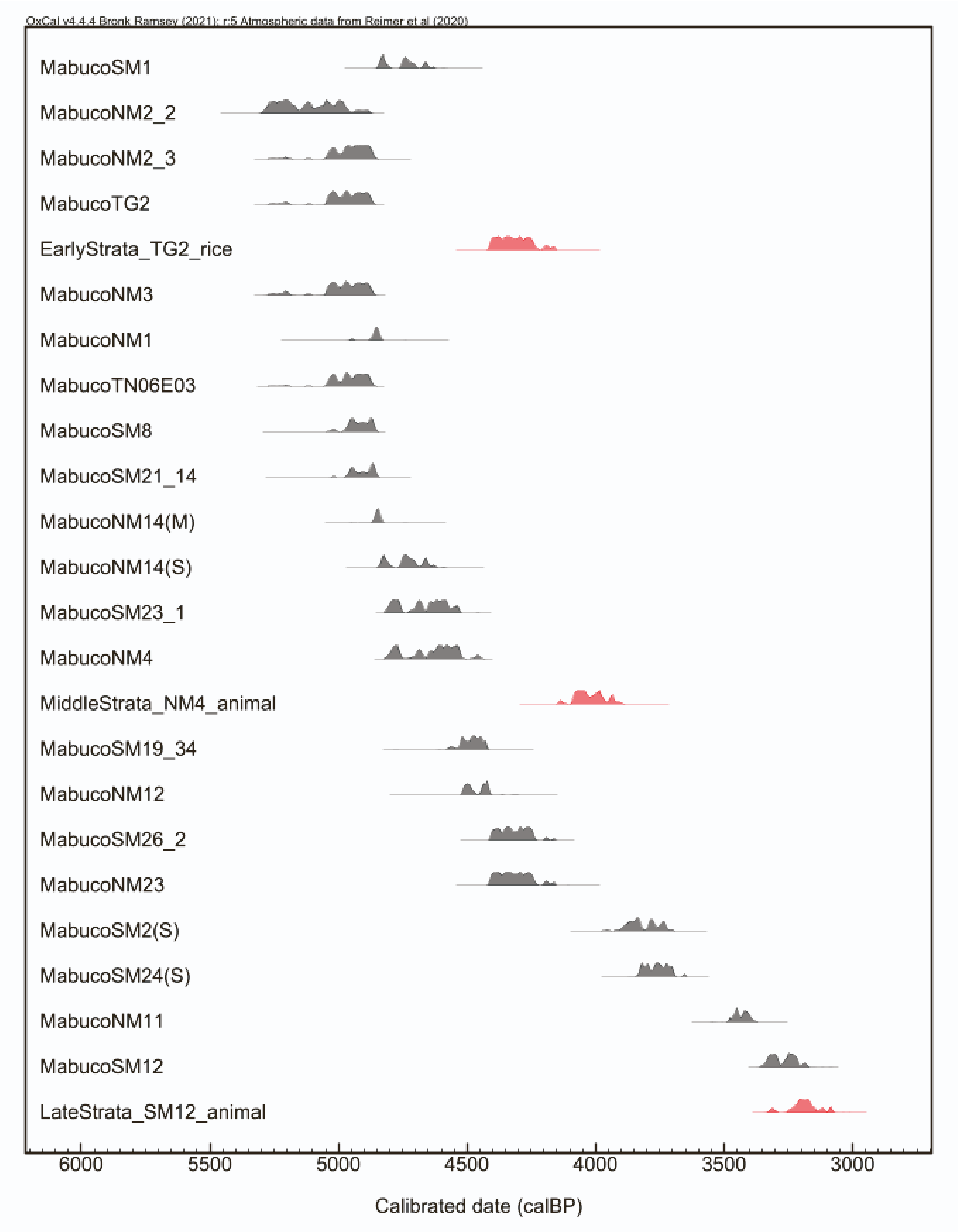

Figure S1. The referenced strata chronology from same archaeological unit with the human bones, related to Figure 1. The dates from the mammal bones and carbonized seeds were younger than the dating result from human bones. The early period starts around 4400 cal BP (with a dating of 3880 ± 30 BP from carbonized rice, 4420–4151 cal BP, 95% CI). The middle period is around 4000 cal BP (with a dating of 3680 ± 30 BP from mammal bones, 4152–3880 cal BP, 95% CI), and the late period is approximately 3500 cal BP (with a dating of 3000 ± 25 BP from mammal bones, 3345–3060 cal BP, 95% CI).

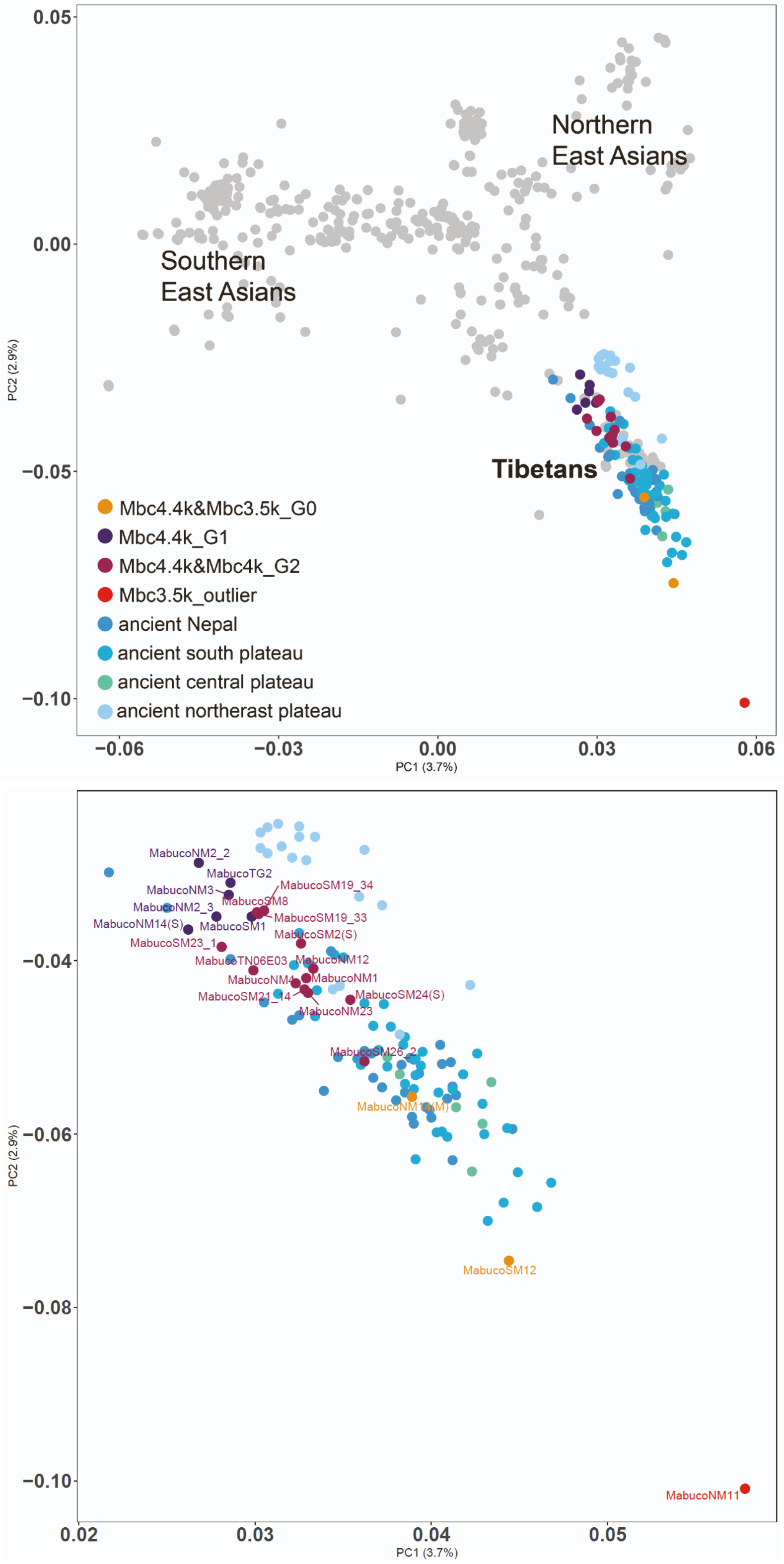

Figure S2. Principal component analysis (PCA) of ancient populations projected onto the PCs calculated by present-day East Asians, related to Figure 2. The upper figure displays the three main clades of populations: northern East Asians, southern East Asians, and Tibetan Plateau populations. The lower figure provides a zoom-in of the Tibetan Plateau populations, with genetic positions of individual samples labeled.

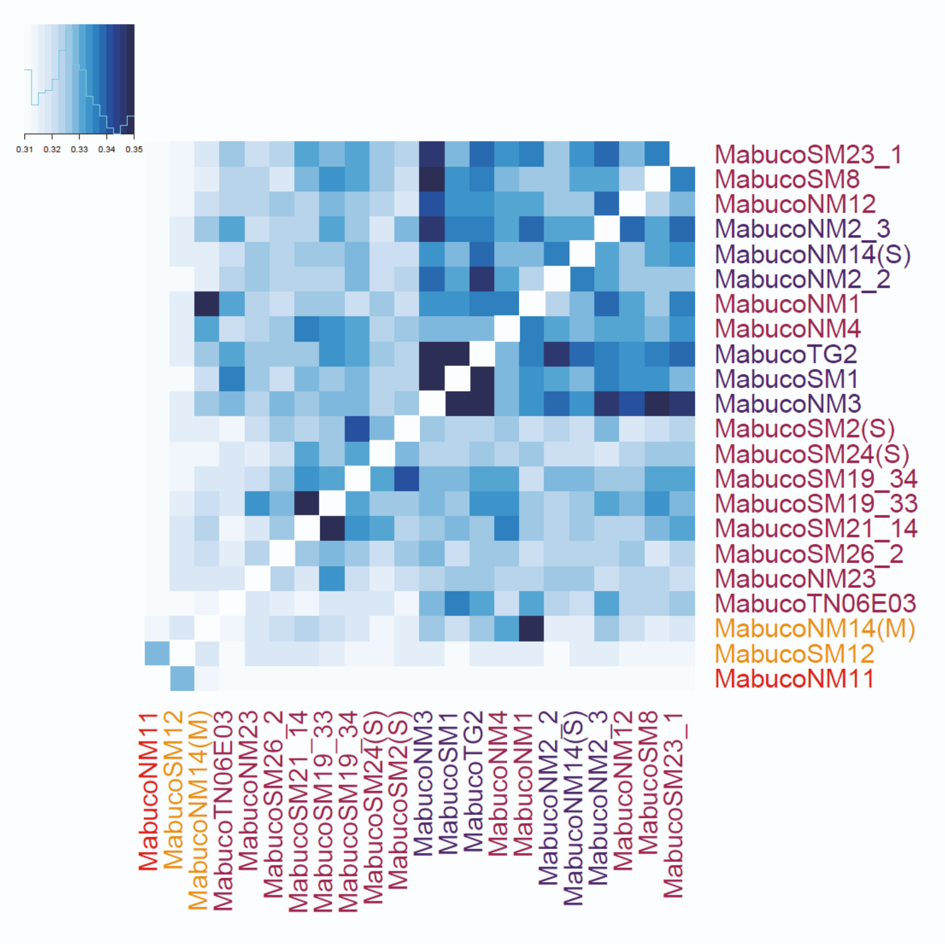

Figure S3. Individual pairwise f3 heatmap, related to Figure 3. F3 statistics (ind1, ind2; Mbuti) showing the genetic connections between pairs of individuals. Darker colors indicate greater shared genetic drift.

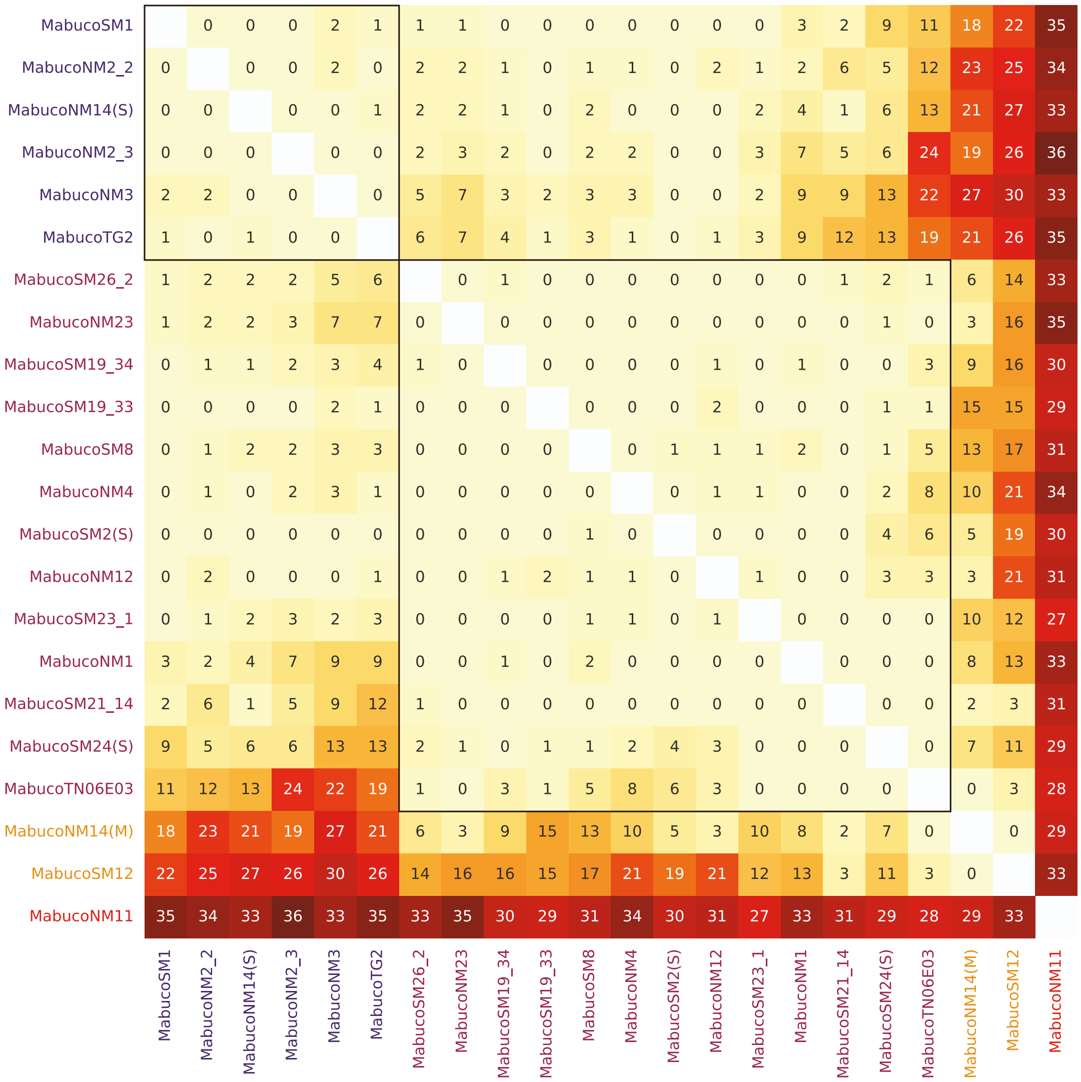

Figure S4. Individual pairwise f4 heatmap, related to Figure 3. Pairwise f4 statistics f4(ind1, ind2; X, Mbuti) assessing genetic affinities among the Mabu Co individuals where X represent worldwide populations (including 54 populations listed as P3 populations in Table S2) used to evaluate differences between pairs of individuals, and the total number of outgroups with significant differences ($|Z| > 3$) is shown in the heatmap, the raw data for plotting can be found in Table S2.

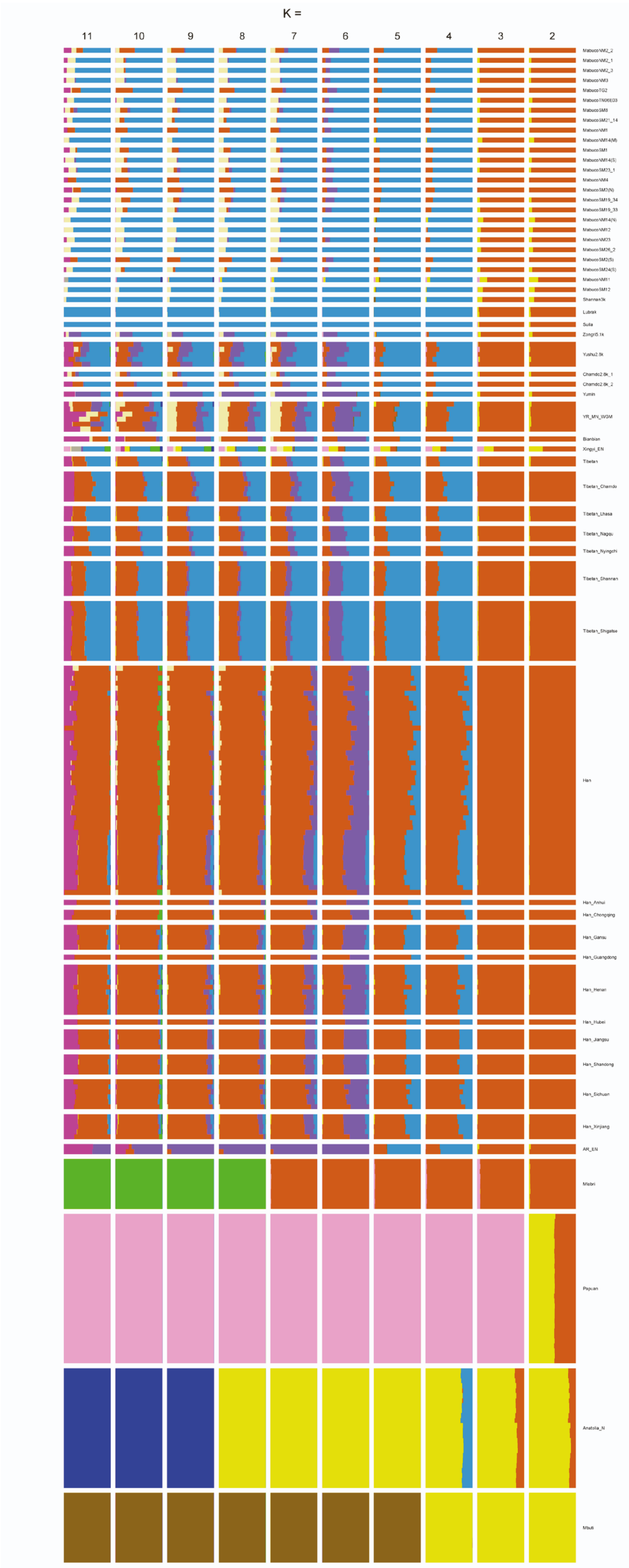

Figure S5 ADMIXTURE displaying ancestry components, related to Figure 3. ADMIXTURE results for selected surrounding populations for K=2 to K=11.

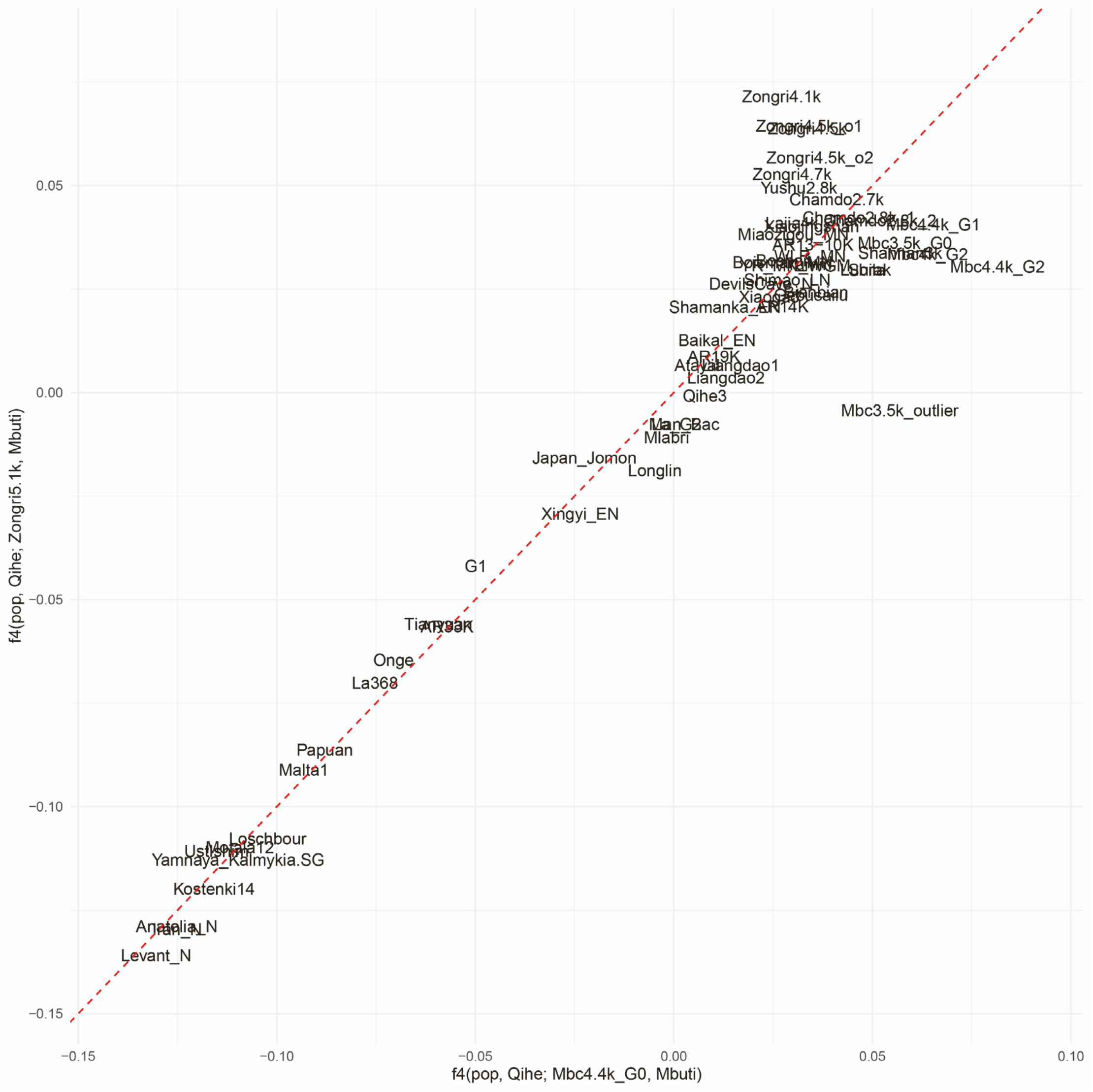

Figure S6. F4 ratio reflect the similarity and differences between Mbc4.4k_G0 and Zongri5.1k, related to Figure 4. The X and Y coordinates of each population point represent the relative genetic drift shared with Mbc4.4k_G0 and Zongri5.1k, respectively, compared to Qihe. The red dashed line (Y = X) indicates f4(pop, Qihe; Mbc4.4k_G0, Mbuti)/f4(pop, Qihe; Zongri5.1k, Mbuti) = 1, where populations equidistant to Mbc4.4k_G0 and Zongri5.1k align with the line. Populations closer to Mbc4.4k_G0 deviate along the X-axis, while those closer to Zongri5.1k align more closely with the Y-axis.

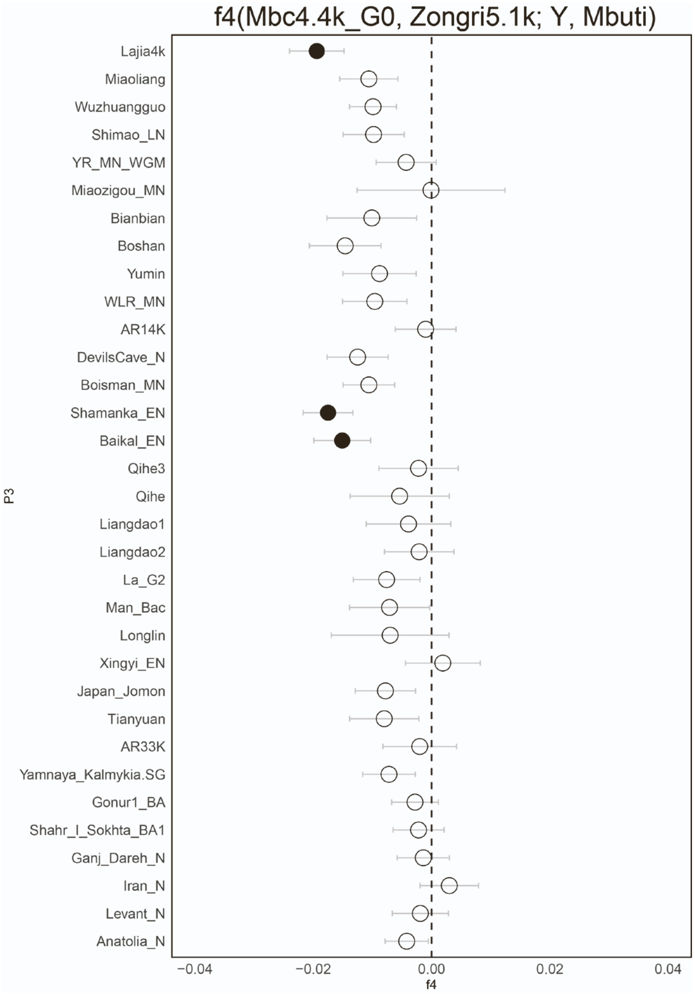

Figure S7. Genetic differences between Mbc4.4k_G0 and Zongri5.1k reflected by f4, related to Figure 4. For the f4(Mbc4.4k_G0, Zongri5.1k; Y, Mbuti) pattern, Y includes non-plateau populations from East Eurasia and Central Asia. Filled circles indicate significant results (Z < -3, suggesting that Y populations share more genetic drift with Zongri5.1k compared to Mbc4.4k_G0). Open circles indicate non-significant results (|Z| < 3). Significant results for populations such as Lajia, Shamanka_EN, and Baikal_EN reveal subtle genetic differences between Mbc4.4k_G0 and Zongri5.1k.

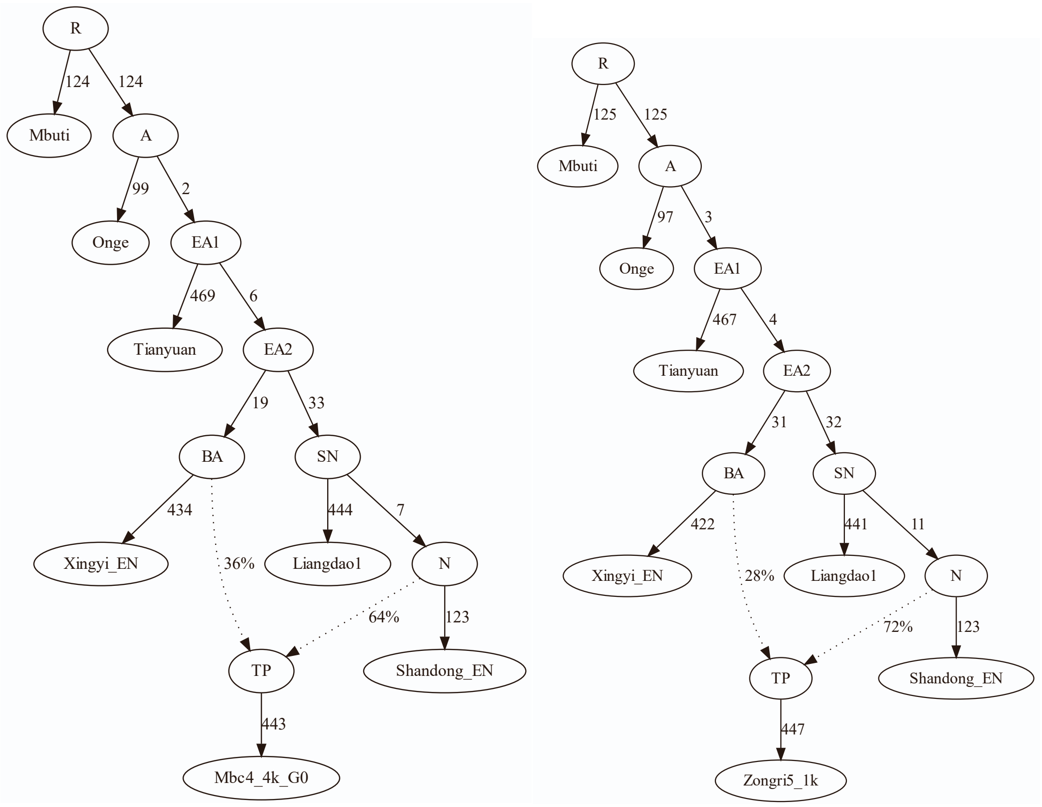

Figure S8. QpGraph modeling of Mbc4.4k_G0 and Zongri5.1k, related to Figure 4. Results of qpGraph estimate that approximately 36% of the ancestry in Mbc4.4k_G0 derives from Basal Asian Xingyi-related ancestry, while the remaining ~64% comes from northern lowland East Asians, consistent with other early Plateau populations.

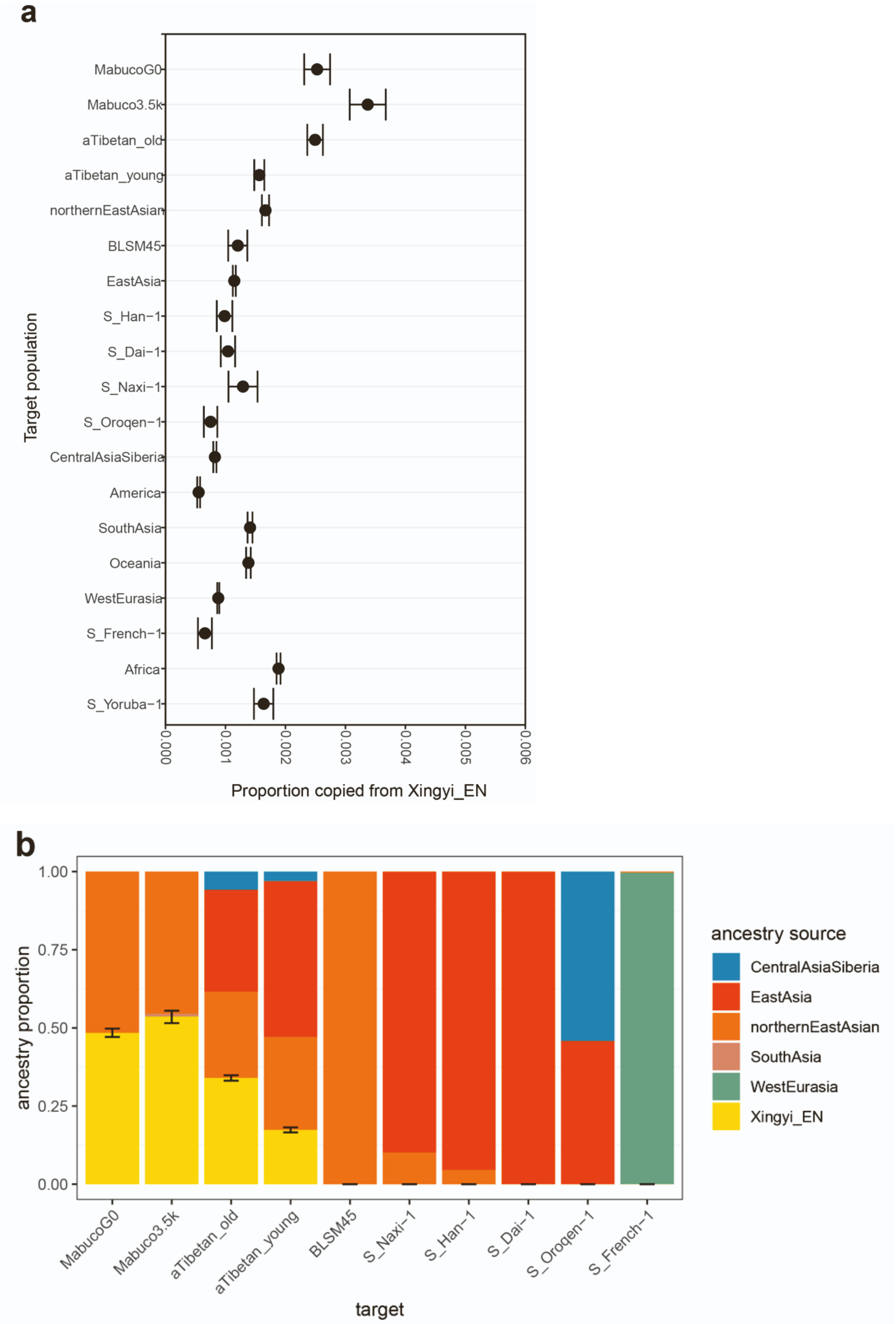

Figure S9. Ancestry estimation using chromosome painting, related to Figure 4. a, Proportions of the genome copied from Xingyi_EN. b, Ancestry proportions inferred by fitting non-negative least squares that model the target painting profiles obtained in b as a mixture of putative source painting profiles. Error bars correspond to the 95% confidence interval of Xingyi_EN-related ancestry obtained from 1000 block bootstrap samples. The set of modern and ancient populations used as references follows Wang et al.[ref 23] to ensure consistency.

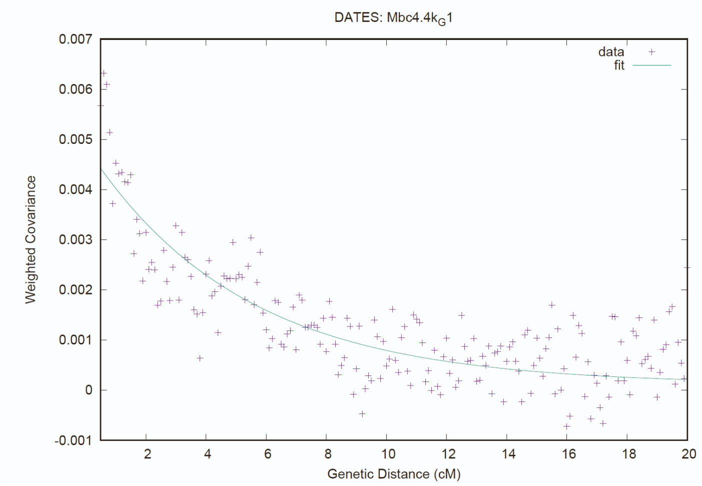

DATES for Mbc4.4k_G1: Mbc4.4k_G0 + preShimao

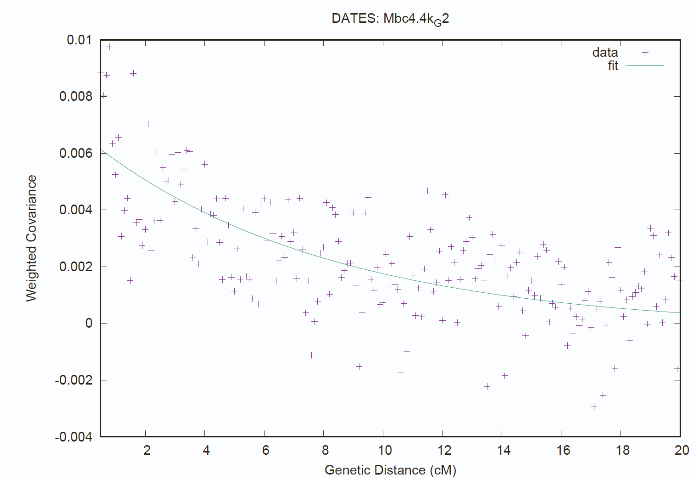

DATES for Mbc4.4k_G2: Mbc4.4k_G0 + YR_MN_WGM

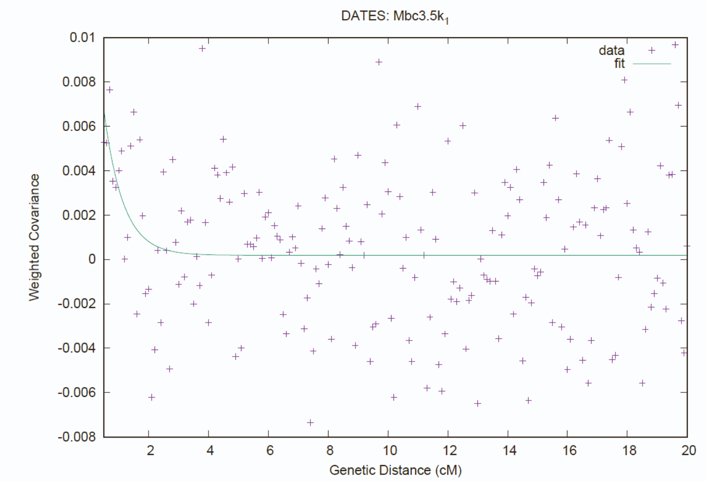

DATES for Mbc3.5k_outlier: Xingyi_EN + Boshan

Figure S10. DATES (Distribution of Ancestry Tracts of Evolutionary Signals) analysis to infer admixture timing, related to Figure 5. Details for Mbc4.4k_G1/G2 and Mbc3.5k_outlier can be found in Table S5 and Table S9, respectively. The image results here meet the simulation parameters thresholds criteria: $Z > 3$, $\lambda < 200$, and normalized root-mean-square deviation (NRMSD) < 0.70.

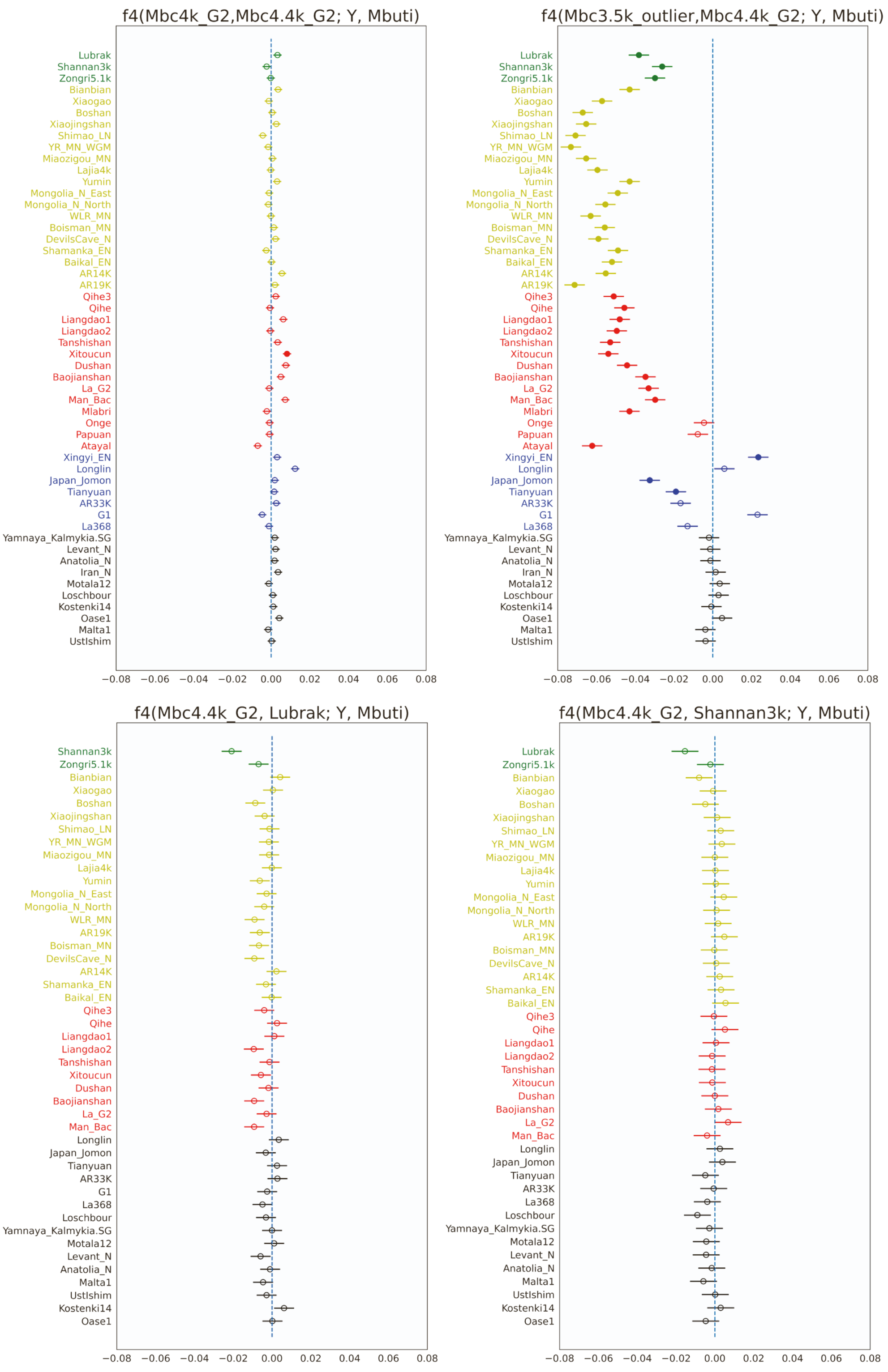

Figure S11. F4 statistics comparing the genetic relationships of Mbc4k_G2, Mbc3.5k_outlier, Lubrak, and Shannan3k with Mbc4.4k_G2, related to Figure 5. The Y-axis includes populations from the early Tibetan Plateau and surrounding regions, filled circles indicate significant results (|Z| > 3), and open circles represent non-significant results (|Z| < 3). For Mbc4k_G2, Lubrak and Shannan3k indicate the genetic continuity of Mbc4.4k_G2, showing no significant differences among them. For Mbc3.5k_outlier, highlights the differences with Mbc4.4k_G2 and close genetic connection to Xingyi_EN.

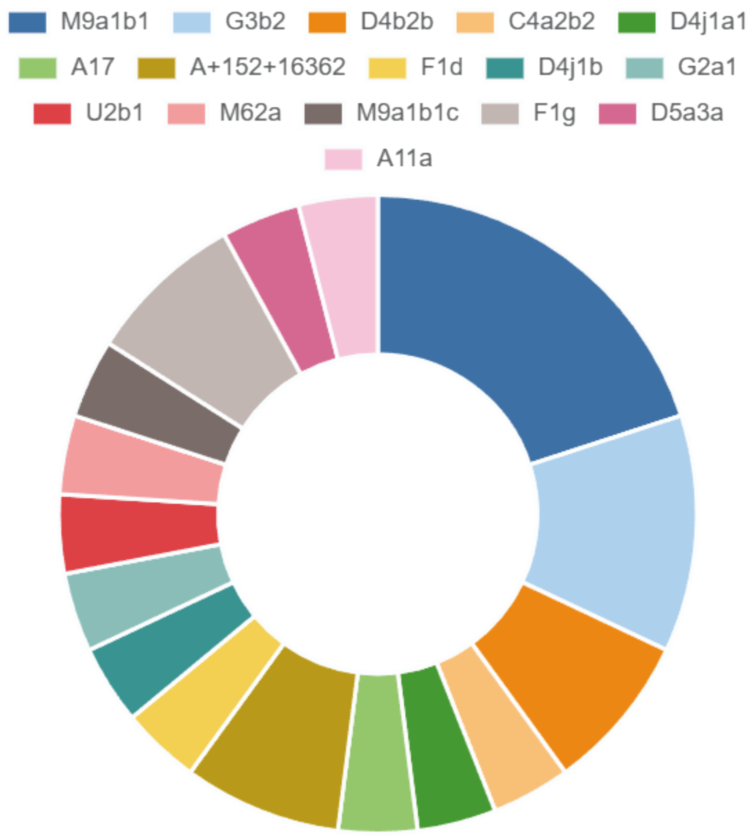

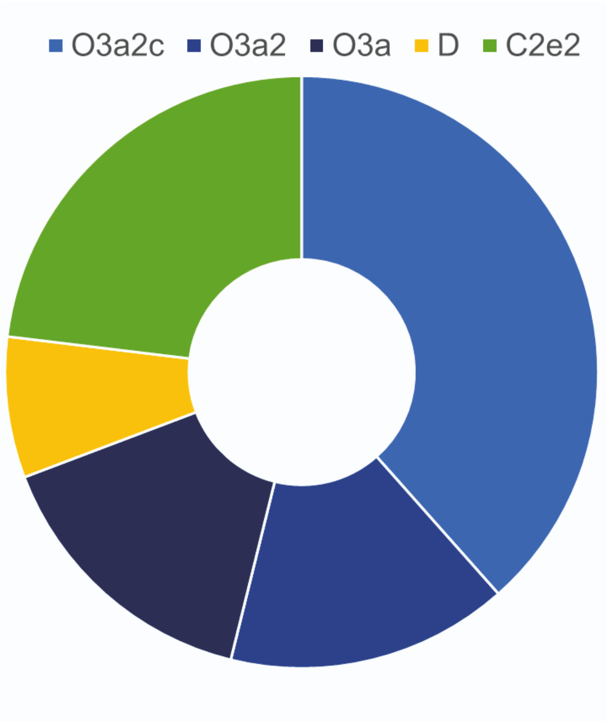

Figure S12. Mitochondrial haplotype assemblies (n=25) and Y chromosome haplotype assemblies (n=13), related to Table S1.
